# Structural and functional characterization of the novel endo-α(1,4)-fucoidanase Mef1 from the marine bacterium *Muricauda eckloniae*

**DOI:** 10.1107/S2059798323008732

**Published:** 2023-10-25

**Authors:** Maria Dalgaard Mikkelsen, Vy Ha Nguyen Tran, Sebastian Meier, Thuan Thi Nguyen, Jesper Holck, Hang Thi Thuy Cao, Tran Thi Thanh Van, Pham Duc Thinh, Anne S. Meyer, Jens Preben Morth

**Affiliations:** a Protein Chemistry and Enzyme Technology Section, Department of Biotechnology and Biomedicine, Technical University of Denmark, DK-2800 Kgs Lyngby, Denmark; b Department of Chemistry, Technical University of Denmark, DK-2800 Kgs Lyngby, Denmark; c NhaTrang Institute of Technology Research and Application, Vietnam Academy of Science and Technology, NhaTrang 650000, Vietnam; Station Biologique de Roscoff, France

**Keywords:** fucoidanases, GH107, crystal structure, (β/α)_8_-barrel, Ca^2+^ site, Mef1, *Muricauda eckloniae*

## Abstract

Fucoidanases (EC 3.2.1.–) catalyze the hydrolysis of glycosidic bonds between fucose residues in fucoidans. Fucoidans are a compositionally and structurally diverse class of fucose-containing sulfated polysaccharides that are primarily found in brown seaweeds. Here, the structural characterization of a novel endo-α(1,4)-fucoidanase, Mef1, from the marine bacterium *Muricauda eckloniae* is presented, showing sequence similarity to members of glycoside hydrolase family 107. Using carbohydrate polyacrylamide gel electrophoresis and nuclear magnetic resonance analyses, it is shown that the fucoidanase Mef1 catalyzes the cleavage of α(1,4)-linkages between fucose residues sulfated on C2 in the structure [-3)-α-l-Fuc*p*2S-(1,4)-α-l-Fuc*p*2S-(1-]_*n*_ in fucoidan from *Fucus evanescens*. Kinetic analysis of Mef1 activity by Fourier transform infrared spectroscopy revealed that the specific Mef1 fucoidanase activity (U_f_) on *F. evanescens* fucoidan was 0.1 × 10^−3^ U_f_ µ*M* ^−1^. By crystal structure determination of Mef1 at 1.8 Å resolution, a single-domain organization comprising a (β/α)_8_-barrel domain was determined. The active site was in an extended, positively charged groove that is likely to be designed to accommodate the binding of the negatively charged, sulfated fucoidan substrate. The active site of Mef1 comprises the amino acids His270 and Asp187, providing acid/base and nucleophile groups, respectively, for the hydrolysis of glycosidic bonds in the fucoidan backbone. Electron densities were identified for two possible Ca^2+^ ions in the enzyme, one of which is partially exposed to the active-site groove, while the other is very tightly coordinated. A water wire was discovered leading from the exterior of the Mef1 enzyme into the active site, passing the tightly coordinated Ca^2+^ site.

## Abstract 2

The first structural determination of the α(1,4)-linkage-specific fucoidan hydrolase Mef1 (GH107) is reported, including the positioning of two calcium sites and the two main amino acids involved in the active-site hydrolytic mechanism. The structural determination also led to the discovery of a water wire leading from the exterior into the active site of the enzyme.

## 1. Introduction

**Figure 1:**
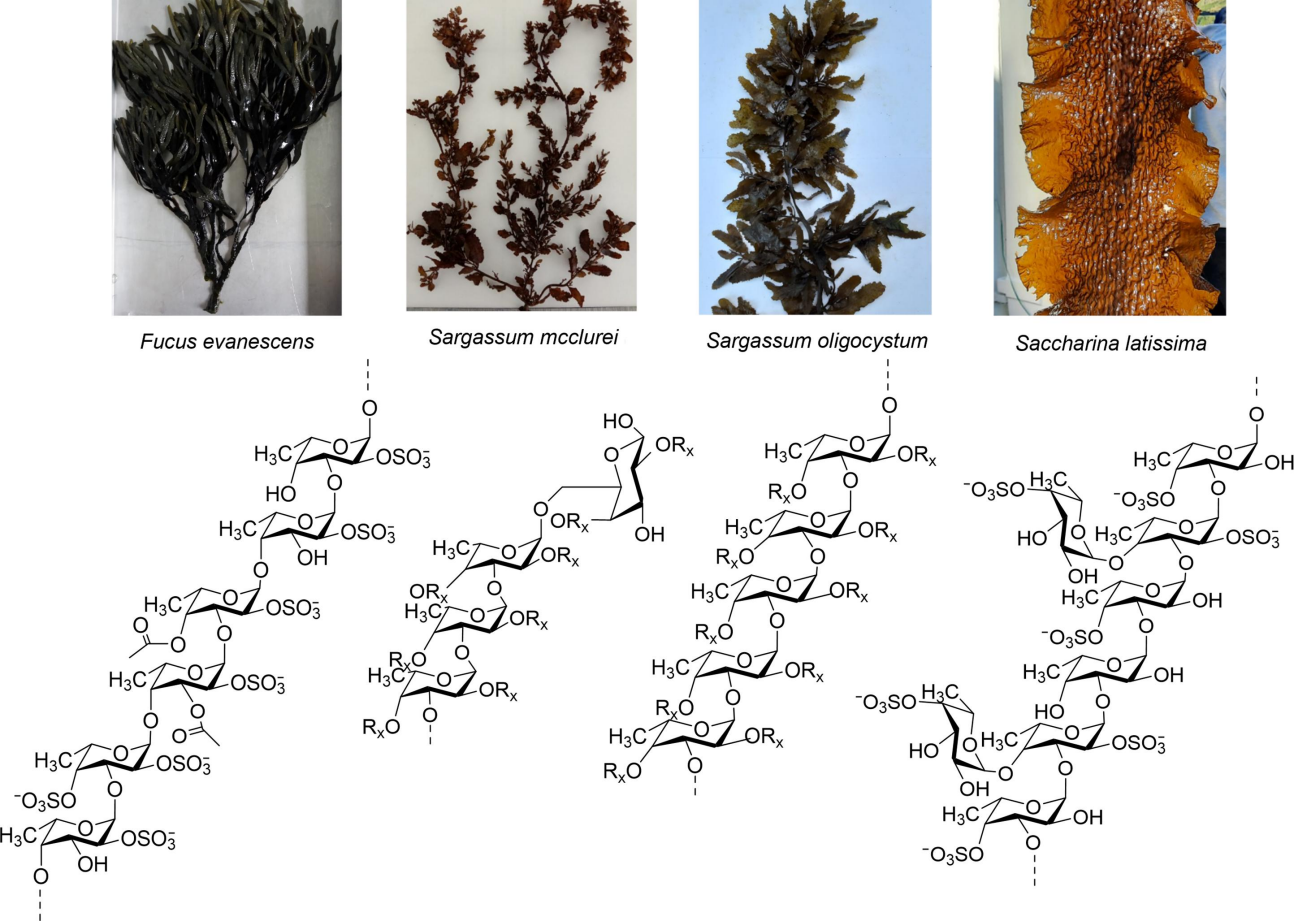
Selected brown seaweeds and the corresponding fucoidans (below) used in the present study. The characteristic structural moieties of the different types of fucoidans corresponding to each type of brown seaweed are indicated . R_x_ indicates H, sulfate, acetate or a side-chain substitution.

Fucoidans are complex sulfated fucose-rich polysaccharides that are primarily derived from brown macroalgae. They are characteristically composed of a backbone of α-linked l-fucose (l-fucosyl or l-fucopyranosyl) moieties with various substitutions (Ale & Meyer, 2013 ▸; Zvyagintseva *et al.*, 2021 ▸). The most well known and most studied fucoidans from brown macroalgae typically have a backbone of alternating α(1→3)- and α(1→4)-linked l-fucosyl moieties. This type is found in the order Fucales, including, for example, *Fucus evanescens*, *F. vesiculosus* and *F. serratus* (Bilan *et al.*, 2002 ▸; Ale & Meyer, 2013 ▸), whereas fucoidans from species of the order Lamin­ariales, for example *Saccharina latissima*, have a backbone composed mainly of α(1→3)-linked l-fucosyl moieties (Bilan *et al.*, 2010 ▸). A third type, known as sulfated galactofucans (Zayed *et al.*, 2022 ▸), are found in various Fucales species prevalent in the Pacific, Western Pacific (South China Sea) and Indian Oceans, for example *Turbinaria ornata*, *Sargassum mcclurei* and *S. oligocystum*, and have a backbone containing both l-fucosyl and d-galactosyl residues (Zvyagintseva *et al.*, 2021 ▸). In all types of fucoidans the l-fucosyl moieties may be substituted with sulfate (– 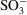 ) or acetate (–COCH_3_) groups and may/or may not have l-fucosyl branches (Fig. 1 ▸).

Fucoidans, including fucoidan oligomers, are known to have biological activities such as anticoagulant, antiviral, anticancer and anti-inflammatory effects (Wang *et al.*, 2019 ▸), and more recently have even been shown to aid in bone regeneration (Nielsen *et al.*, 2022 ▸; Ohmes *et al.*, 2022 ▸), making them interesting as potential biomedicals. Fucoidans are used as dietary supplements and in cosmetics, and can be degraded to lower molecular-weight oligomers using acids or oxidants such as hydrogen peroxide to achieve improved bioactivity (Lahrsen *et al.*, 2018 ▸). However, because of the nonspecific cleavage of the fucoidan molecules by such treatments, the products of these treatments are heterogeneous, which is a barrier to obtaining regulatory approval for clinical pharmaceutical purposes.

Fucoidanases, including endo-α(1,3)-fucoidanase (endo-α-1,3-l-fucanase; EC 3.2.1.211) and endo-α(1,4)-fucoidanase (endo-α-1,4-l-fucanase; EC 3.2.1.212), are glycoside hydrolases that are active only on fucoidan substrates containing the characteristic target glycosidic linkage, sulfation pattern and acetylation. Enzymatic hydrolysis will therefore result in the release of a population of homogeneous fucoidan oligosaccharides, the chemical structures of which are dependent on the specific fucoidanase and fucoidan substrate.

Based on sequence classification, the endo-α(1,4)-l-fucoid­anases (EC 3.2.1.212) belong to glycoside hydrolase family 107 (GH107) in the CAZy categorization and the endo-α(1,3)-l-fucoidanases (EC 3.2.1.211) belong to GH168, with some categorized into the recently established GH174 (Drula *et al.*, 2022 ▸). Recently, an endo-α(1,3)-fucoidanase (EC 3.2.1.211) activity has been added to the GH107 family by the characterization of the fucoidanase Mef2 (Tran, Nguyen *et al.*, 2022 ▸), which originates from *Muricauda eckloniae* like the fucoidanase Mef1 studied in the present paper.

The first characterized GH107 endo-α(1,4)-fucoidanase was MfFcnA from the marine bacterium *Mariniflexile fucanivorans* (Colin *et al.*, 2006 ▸). More recently, other GH107 endo-α(1,4)-fucoidanases have been characterized, including, for example, Mef2 from *M. eckloniae*, which was functionally characterized as a family GH107 endo-α(1,3)-fucoidanase (Tran, Nguyen *et al.*, 2022 ▸), and Fhf1 and Fhf2 from the marine bacterium *Formosa haliotis* (Vuillemin *et al.*, 2020 ▸; Trang *et al.*, 2022 ▸). C-terminal deletion mutants of Fhf1 (Fhf1Δ470; Vuillemin *et al.*, 2020 ▸) and Fhf2 (Fhf2Δ484;Trang *et al.*, 2022 ▸) were found to be poly[α(1,4)-l-fucoside-2S] glycoside hydrolases that catalyze the cleavage of the structural motif [-3)-α-l-Fuc*p*2S-(1,4)-α-l-Fuc*p*2S-(1-] of fucoidans isolated from *F. evanescens*, but Fhf2 also released longer oligosaccharides with disulfations (Trang *et al.*, 2022 ▸).

To date, MfFcnA and *Psychromonas* P5AFcnA are the only fucoidanases that have been structurally determined at high resolution (Vickers *et al.*, 2018 ▸). The structure of P5AFcnA was determined at 1.55 Å resolution and was found to consist of only a (β/α)_8_-barrel domain, identified as the catalytic D1 domain, containing a single Ca^2+^ ion (Vickers *et al.*, 2018 ▸). The 2.2 Å resolution crystal structure of MfFcnA exhibited a four-domain organization, including a D1 domain with a fold similar to that of P5AFcnA and in addition three predicted Ig-like domains (IgR domains) at the C-terminal end. MfFcnA was found to contain five Ca^2+^ ions (Ca1–Ca5), with Ca1 and Ca2 in the catalytic D1 domain and the other three (Ca3–Ca5) in the IgR domains (Vickers *et al.*, 2018 ▸).

In the present study, we describe the reaction selectivity and the detailed structure of the novel GH107 endo-fucoidanase Mef1 from the marine bacterium *M. eckloniae* for the first time. The structure of Mef1 was determined by X-ray crystallography, while characterization of the reaction was performed by monitoring the reaction using real-time nuclear magnetic resonance (NMR) and Fourier transform infrared spectroscopy (FTIR).

## 2. Materials and methods

### 2.1. Materials

Fucoidan from *F. vesiculosus* was purchased from Sigma–Aldrich, Steinheim, Germany. Fucoidans from *F. evanescens* (seaweed was harvested in the Kiel Canal and kindly provided by Coastal Research & Management, Kiel, Germany) and *S. latissima* (cultivated *S. latissima* seaweed was kindly provided by Ocean Rainforest, Kaldbak, Faroe Islands) were extracted using an enzyme-assisted method and fractionated by ion-exchange chromatography using DEAE resin as described previously (Nguyen *et al.*, 2020 ▸). Fucoidans from *Sargassum mcclurei*, *Turbinaria ornata*, *S. polycystum*, *Hormophysa cuneiformis*, *S. oligocystum* and *S. serratum* (all seaweeds were collected in NhaTrang Bay, Vietnam by trained staff from the NhaTrang Institute of Technology Research and Application, Vietnam) were extracted by a chemical method as described previously (Bilan *et al.*, 2002 ▸) and fractionated by ion-exchange chromatography using DEAE resin (Nguyen *et al.*, 2020 ▸). Monomeric composition analysis of the fucoidan substrates was performed as described previously (Manns *et al.*, 2014 ▸; Nguyen *et al.*, 2020 ▸). The hydrolysable fucoidan substrate compositions are given in Supplementary Table S1. For *F. evanescens* fucoidan, fraction 3 was used as substrate for enzyme assays, while fraction 4 was used for FTIR experiments (Tran, Perna *et al.*, 2022 ▸); the monosaccharide compositions of these two *F. evanescens* fucoidan fractions are given in Supplementary Table S2 (analyzed as described previously; Nguyen *et al.*, 2020 ▸).

### 2.2. High-performance size-exclusion chromatography (HP-SEC) analysis of fucoidans

The size of the fucoidan substrates and Mef1-hydrolyzed products were determined by HP-SEC using an Ultimate iso-3100SD pump with a WPS-3000 sampler (Dionex, Sunnyvale, California, USA) connected to an RI-101 refractive-index (RI) detector (Shodex, Showa Denko K.K., Tokyo, Japan). Samples were separated on a Shodex SB-806 HQ GPC column (300 × 8 mm) equipped with a Shodex SB-G guard column (50 × 6 mm) with elution using 100 m*M* sodium acetate buffer pH 6 at a flow rate of 0.5 ml min^−1^ at 40°C (Trang *et al.*, 2022 ▸). Pullulans of 1, 5, 12, 110, 400 and 800 kDa (Sigma–Aldrich, Steinheim, Germany) were used as standards.

### 2.3. Sequence analysis

The putative fucoidanase Mef1 (GenBank AAY42_01290; KQC28683.1) was identified using *BLASTp* analysis against a set of GH107 endo-fucoidanases. The N-terminal signal peptide was predicted using the *SignalP* 5.0 server (https://services.healthtech.dtu.dk/services/SignalP-5.0/). Protein sequence comparisons were performed using *Clustal Omega* (https://www.ebi.ac.uk/Tools/msa/clustalo/) for global alignments. The endo-fucoidanases P5AfcnA (GenBank AYF59291.1, *Psychromonas* sp. SW5A, phylum Pseudomonadota), P19DfcnA (GenBank AYF59292.1, *Psychromonas* sp. SW19D, phylum Pseudomonadota), FWf3 (GenBank ANW96115.1, *Wenyingzhuangia fucanilytica* CZ1127, phylum Bacteroidota), Fp273 (GenBank AYC81238.1, uncultured bacterium, phylum unknown), FFA2 (RefSeq WP_057784219.1, *Formosa algae*, phylum Bacteroidota), Fhf2 (GenBank UQB70640.1 and UQB70641.1, *F. haliotis*, phylum Bacteroidota), Fhf1 (GenBank UQB70638.1 and UQB70639.1, *F. haliotis*, phylum Bacteroidota), FWf4 (GenBank ANW96116.1, *W. fucanilytica* CZ1127, phylum Bacteroidota), MfFcnA (GenBank CAI47003.1, *Mariniflexile fucanivorans*, phylum Bacteroidota), FFA1 (RefSeq WP_057784217.1, *F. algae*, phylum Bacteroidota), FWf2 (GenBank ANW96098.1, *W. fucanilytica* CZ1127, phylum Bacteroidota), Fp279 (GenBank AYC81240.1, uncultured bacterium, phylum unknown), FWf1 (GenBank ANW96097.1, *W. fucanilytica* CZ1127, phylum Bacteroidota), Fp277 (GenBank AYC81239.1, uncultured bacterium, phylum unknown), SVI_0379 (GenBank BAJ00350.1, *Shewanella violacea* DSS12, phylum Pseudomonadota), Fda1 (GenBank AAO00508.1, organism unknown, phylum unknown), Fda2 (GenBank AAO00509.1, organism unknown, phylum unknown) and Mef2 (GenBank URS64324.1; *Muricauda eckloniae*, phylum Bacteroidota) were used for sequence comparisons.

Conserved residues of the catalytic domain were identified by multiple sequence alignment using *MAFFT* and visualized using *Jalview* (EMBL_EBI; https://www.ebi.ac.uk/Tools/msa/mafft/). *ESPript* 3.0 (https://espript.ibcp.fr/ESPript/ESPript/; Robert & Gouet, 2014 ▸) was used to visualize the alignment and to incorporate α-helices and β-strands from the crystal structure data (Mef1, PDB entry 8bpd; P5AFcnA, PDB entry 6m8n) for comparisons.

For the phylogenetic analysis, the sequences were aligned using *Muscle* v.3.7 with default settings, with the removal of gaps. The curated sequences were used to build a maximum-likelihood phylogenetic tree using *phyML* with default settings. The phylogenetic tree was statistically supported by approximate likelihood-ratio testing using default settings and values between 0 and 1 were obtained together with bootstrap values using https://www.phylogeny.fr. *FigTree* was used for visualization and fine adjustments of the tree. Protein domains were predicted using *InterProscan* (http://www.ebi.ac.uk/interpro/search/sequence/)

### 2.4. Gene construction and cloning

The Mef1 gene construct was designed without the N-terminal signal peptide but with a C-terminal 10×His tag. The Mef1 fucoidanase was codon-optimized for *Escherichia coli* expression and subcloned into the pET-28b(+) vector between the NheI and XhoI restriction sites by GenScript, Piscataway, New Jersey, USA. The construct (GenBank OQ831695) was transformed into *E. coli* strain DH5α (Thermo Fisher Scientific, Waltham, Massachusetts, USA) for plasmid propagation using LB (lysogeny broth) agar plates with kanamycin and incubation at 37°C overnight.

### 2.5. Recombinant enzyme expression and purification

Expression of the fucoidanase Mef1 was performed in *E. coli* BL21 (DE3) cells harboring the Pch2 (pGro7) plasmid (Takara Biolabs, Göteborg, Sweden). For expression, LB medium with 50 µg ml^−1^ kanamycin, 34 µg ml^−1^ chloram­phenicol and 0.05%(*w*/*v*) arabinose was inoculated with an overnight culture in the same medium and incubated at 37°C and 180 rev min^−1^. Once the culture reached an OD_600_ of 0.6–0.8, it was cooled to 20°C and incubated at this temperature for 2 h at 180 rev min^−1^ before adding 1 m*M* isopropyl β-d-1-thiogalactopyranoside (IPTG), after which the culture was incubated overnight at 20°C and 180 rev min^−1^. The cells were collected by centrifugation (10 000*g*, 4°C, 20 min) and the cell pellets were stored at −80°C. After thawing, the cells were resuspended in 20 m*M* Tris–HCl buffer, 250 m*M* NaCl, 20 m*M* imidazole pH 7.4. The cells were then lysed on ice by three cycles of sonication (P400S Ultrasonic processor; Hielscher, Teltow, Germany) in the presence of 0.2 mg ml^−1^ lysozyme (Sigma–Aldrich, Steinheim, Germany). After centrifugation (19 000*g*, 4°C, 20 min) the supernatant was filtered (0.22 µm) and mixed with Ni^2+^–NTA Sepharose resin (GE Healthcare, Uppsala, Sweden) pre-equilibrated with equilibration buffer (20 m*M* Tris–HCl buffer, 250 m*M* NaCl, 20 m*M* imidazole pH 7.4). To maximize binding, the resin suspension was incubated at 4°C overnight with gentle agitation. The resin was then washed three times with buffer (20 m*M* Tris–HCl buffer pH 7.4, 250 m*M* NaCl, 20 m*M* imidazole) and packed onto a column. The recombinant protein was eluted with 20 m*M* Tris–HCl buffer, 250 m*M* NaCl pH 7.4 and increasing concentrations of imidazole (100–500 m*M*). Fractions containing the pure enzyme were pooled and desalted using PD-10 columns (GE Healthcare, Uppsala, Sweden) equilibrated with 20 m*M* Tris–HCl buffer, 100 m*M* NaCl pH 8.0 to remove the imidazole. Protein concentration was measured by the Bradford assay using bovine serum albumin as a standard (Bradford, 1976 ▸). The purified enzyme was stored at −80°C.

### 2.6. SDS–PAGE and Western blot

The purity and molecular weight of the recombinantly expressed proteins were assessed by sodium dodecyl sulfate–polyacrylamide gel electrophoresis (SDS–PAGE) using 12% acrylamide gels according to the Laemmli protocol (Laemmli, 1970 ▸). After mixing with 1 m*M* dithiothreitol and Laemmli loading buffer (Bio-Rad, Hercules, California, USA), proteins were incubated at 95°C for ∼10 min. The Precision Plus Protein Standards (Unstained) molecular-weight marker (Bio-Rad) with molecular weights of 10–250 kDa was used as a standard ladder. Gels were stained using Coomassie Brilliant Blue (Bio-Rad). After separation by SDS–PAGE, proteins were transferred onto a PVDF blotting membrane (GE Healthcare, Chicago, Illinois, USA) which had been pre-wetted with ethanol for a few seconds, and blotting was then performed in Tris–glycine running buffer pH 8.3 at 100 V for 45 min on ice. After protein transfer, the membrane was blocked with 2% skimmed milk in 0.01 *M* Tris base sodium chloride pH 7.6 (TBS) buffer for 60 min and then incubated with monoclonal anti-polyhistidine peroxidase-conjugated antibody (1:10 000 dilution; Sigma–Aldrich, Steinheim, Germany) for another 60 min. The membrane was washed in TBS buffer three times for 20 min, and the Western blot was developed by staining with the AEC Kit following the manufacturer’s protocol (Sigma–Aldrich). Precision Plus Protein Dual Color Standard (Bio-Rad) was used as the molecular-weight marker.

### 2.7. Endo-fucoidanase activity assay by C-PAGE

The fucoidanase activity was monitored using carbohydrate polyacrylamide gel electrophoresis (C-PAGE) analysis. The reaction consisted of 0.12 mg ml^−1^ Mef1 enzyme, 0.02 *M* Tris–HCl buffer pH 8.0, 50 m*M* NaCl, 0.9 mg ml^−1^ fucoidan and 10 m*M* CaCl_2_ at 37°C. To ensure significant fucoidan degradation, the reaction was performed for 24 h. As a standard, fucoidan from *F. evanescens* was hydrolyzed by FFA2 (Silchenko *et al.*, 2017 ▸) as described previously (Vuillemin *et al.*, 2020 ▸). Loading buffer [20%(*v*/*v*) glycerol and 0.02%(*w*/*v*) phenol red in a 1:1 ratio] was added to all samples before loading onto the C-PAGE gel. C-PAGE was run on a 20%(*w*/*v*) acrylamide/bisacrylamide electrophoresis gel with 100 m*M* Tris–borate buffer pH 8.3 for 90 min at 30 mA. Gel staining was performed in a solution consisting of 0.05% Alcian blue 8GX (Panreac, Spain) in 2% acetic acid and 0.01% *O*-toluidine blue (Sigma–Aldrich).

### 2.8. Endo-fucoidanase activity assay by Fourier transform infrared spectroscopy (FTIR)

Spectral evolution profiles were achieved by FTIR measurements on enzyme reaction mixtures consisting of 2%(*w*/*v*) fucoidan from *F. evanescens* in 20 m*M* Tris–HCl buffer pH 8.0, 100 m*M* NaCl, 10 m*M* CaCl_2_ at 40°C according to Tran, Perna *et al.* (2022 ▸). The enzyme was dosed at different levels to attain measurable spectra within 2 h. After addition of the enzyme, the reaction mixture was immediately injected into the FTIR instrument (MilkoScan FT2, FOSS Analytical, Hillerød, Denmark) and up to 400 spectra were acquired consecutively during the reaction. The spectral evolution profiles of the enzyme reactions were analyzed by parallel factor analysis (PARAFAC; Harshman & Lundy, 1994 ▸) and the linear fits of the PARAFAC models were used to quantify the enzyme activity from the continuous reaction data (Tran, Perna *et al.* 2022 ▸).

### 2.9. Characterization of recombinantly expressed Mef1 using C-PAGE

Substrate-specificity screening experiments were performed using different fucoidan substrates at 0.9 mg ml^−1^, 10 m*M* CaCl_2_ and 0.12 mg ml^−1^ purified Mef1 in 20 m*M* Tris–HCl pH 8.0, 50 m*M* NaCl at 37°C overnight. The biochemical characteristics of the Mef1 fucoidanase were determined using 0.12 mg ml^−1^ enzyme in 20 m*M* Tris–HCl, 50 m*M* NaCl incubated with 10 mg ml^−1^ substrate for 2 h at 37°C. The influence of different temperatures (20–70°C) on fucoidanase activity was studied by changing the assay temperature. Likewise, the influence of NaCl concentration (25–500 m*M*) on fucoidanase activity was investigated at different levels of NaCl addition to the assay.

The influence of different buffers (Tris–HCl, borate, phosphate and UB4 buffer) at pH 8.0 was investigated at 0.02 *M* buffer concentration. The influence of pH (4–10) on fucoidanase activity was examined using buffers with different pH values (0.02 *M* UB4 buffer with a pH between 4.0 and 8.0, 0.02 *M* borate buffer with a pH between 8.0 and 10.0 or 0.02 *M* Tris–HCl with a pH between 7.0 and 8.5).

The influence of divalent cations (Ca^2+^, Mg^2+^, Mn^2+^, Cu^2+^, Fe^2+^, Zn^2+^, Co^2+^ and Ni^2+^) on activity was investigated by the addition of 10 m*M* of each cation after treatment with EDTA (2 m*M*) and EDTA removal by treatment with PD10. Time-course experiments were performed using 0.12 mg ml^−1^ Mef1 in 0.02 *M* Tris–HCl buffer pH 8.0, 50 m*M* NaCl, 10 m*M* CaCl_2_ and 0.9 mg ml^−1^ fucoidan from *F. evanescens*. The reactions were incubated for 48 h. All assays were performed for 2 h unless stated otherwise. The results were visualized by C-PAGE.

### 2.10. Thermal stability of the recombinant fucoidanase Mef1

Differential scanning fluorimetry (DSF) was carried out to determine the thermal stability of the Mef1 protein. The concentration of Mef1 (5 µ*M*) was determined by measuring the absorbance at 280 nm using a calculated molar extinction coefficient computed using *ProtParam* from ExPASy. Samples were added to 10 µl capillary tubes and analyzed using a Prometheus NT.Plex nanoDSF instrument (Nanotemper Technologies, Munich, Germany). Thermal stability was monitored using a temperature gradient of 25–80°C with an increase of 1°C min^−1^ to obtain denaturation profiles. Raw data were exported into data sets that contained fluorescence at 330 and 350 nm (*F*_330_ and *F*_350_) as well as the ratio of these values (*F*_330_/*F*_350_) and absorbance at 350 nm (*A*_350_). The first derivatives of *F*_330_/*F*_350_ were plotted to visualize denaturation. The peak of the first derivative gives the value of the melting temperature (*T*_m_), at which half of the protein is unfolded.

### 2.11. Preparation and isolation of enzymatic hydrolysis products

The reaction mixture consisted of 0.12 mg ml^−1^ Mef1 in 0.02 *M* Tris–HCl buffer pH 8.0 containing 50 m*M* NaCl, 10 m*M* CaCl_2_ and 0.9 mg ml^−1^ fucoidan from *F. evanescens*. Reactions were run at 37°C for 24 h and stopped by heating at 80°C for 10 min. The precipitated proteins were removed by centrifugation at 19 000*g* for 20 min. Cold ethanol was added to the reaction mixture to a final concentration of 72%(*v*/*v*) to precipitate the medium-molecular-weight fucoidan products (MMP) and in this way separate them from the low-molecular-weight fucoidans (LMP), followed by centrifugation at 19 000*g* for 20 min. The supernatant containing LMP was evaporated under an air flow. Both MMP and LMP were solubilized in distilled water and lyophilized. The products were analyzed by C-PAGE and NMR spectroscopy; the yields and monosaccharide compositions of MMP and LMP are shown in Supplementary Table S3 (compositional analysis was performed as described in Nguyen *et al.*, 2020 ▸).

### 2.12. NMR spectroscopy

Fucoidans from *F. evanescens*, including native fucoidan and the reaction products (LMP and MMP), were dissolved in 500 µl ^2^H_2_O. All NMR spectra were collected using an 800 MHz Bruker Avance III instrument equipped with a TCI cryoprobe (5 mm) by acquiring ^1^H–^1^H TOCSY (2048 × 256 complex data points sampling 128 and 16 ms in the direct and indirect dimensions, respectively, and using a 10 kHz spin-lock field), ^1^H–^1^H COSY (2048 × 256 complex data points sampling 128 and 16 ms in the direct and indirect dimensions, respectively), ^1^H–^13^C HMBC (2048 × 128 complex data points sampling 256 and 6.3 ms) and ^1^H–^13^C HSQC (2048 × 512 complex data points sampling 160 and 21.2 ms).

The time-dependent degradation of *F. evanescens* fucoidan by Mef1 was followed using 0.005 mg ml^−1^ enzyme and 0.9% substrate in 10 m*M* Tris–HCl buffer pH 8. A time series of ^1^H–^13^C HSQC spectra (2048 × 128 complex data points sampling 212 and 5.3 ms in the ^1^H and ^13^C dimensions, respectively) was acquired at 25°C with a non-uniform sampling of 30% of the data points in the indirect dimension. All NMR spectra were processed with ample zero filling and baseline corrections in all dimensions using Bruker *TopSpin* 3.5 pl7 and were analyzed using the same software.

### 2.13. Size-exclusion chromatography of Mef1

Analytical size-exclusion chromatography (SEC) was used to confirm the molecular size of the protein and the state of oligomerization. SEC was performed using 200 µl enzyme (at 2 mg ml^−1^) with elution in 20 m*M* degassed Tris–HCl pH 8.0 containing 50 m*M* NaCl on a chromatography system using a high-resolution Superdex 200 10/300 GL column and a flow rate of 0.75 ml min^−1^. Mef1 was detected by UV absorbance at 280 nm.

### 2.14. X-ray crystallography, structure refinement and molecular docking of Mef1

Purified Mef1 protein after SEC was concentrated to 16 mg ml^−1^ using Pierce Protein Concentrators PES 3K MWCO (Thermo Fisher, Waltham, Massachusetts, USA). Crystallization trials of Mef1 were performed at 18°C using the vapor-diffusion method in sitting drops consisting of a 1:1 ratio of pure protein (16 mg ml^−1^ in 20 m*M* Tris–HCl pH 8.0, 50 m*M* NaCl) and precipitant solution (the LMB Crystallization Screen from Molecular Dimensions and Index from Hampton Research) in the presence or absence of divalent ions (1 m*M* Ca^2+^ or 1 m*M* Cu^2+^). After obtaining initial Mef1 crystals using 18% PEG 3350, 0.1 *M* 3-(cyclohexyl­amino)-2-hydroxy-1-propane sulfonic acid (CAPSO) pH 9, 4.8% 2-propanol, 17% PEG 400, the conditions for crystallization were optimized using grid optimization screens in 24-well sitting drops at 18°C using the hanging-drop vapor-diffusion method. The optimized parameters included screening of the 2-(cyclohexylamino)ethanesulfonic acid (CHES) and CAPSO concentrations, different concentrations of the enzyme (10, 12 and 16 mg ml^−1^) and three different dilutions (1:2, 2:1 and 1:1) of protein and crystallization solution (total volume 2 µl).

Optimal crystals of Mef1 were obtained using 12 mg ml^−1^ Mef1, 1 m*M* CaCl_2_, 50 m*M* NaCl, 20 m*M* Tris pH 8.0 in a drop consisting of 24% PEG 3350, 0.1 *M* CAPSO pH 9, 3% 2-propanol, 4% PEG 400. The crystals were mounted and soaked in 18% ethylene glycerol and flash-cooled to cryotemperatures using liquid nitrogen.

X-ray diffraction data collection was performed on the BioMax beamline at MAX IV, Lund, Sweden. The beamline features a PILATUS 6M detector. Data were collected at 100 K for a full sweep of 360° with 0.1° oscillation and 0.050 s exposure time at 12.6696 keV. A complete data set was processed from 120° of data (1200 images). Data were collected in space group *P*6_3_ at 1.8 Å resolution. The structure of Mef1 was determined by molecular replacement with *Phaser-MR* (McCoy *et al.*, 2007 ▸) using the homologous GH107 endo-fucoidanase P5AFcnA as a search model (PDB entry 6m8n; Vickers *et al.*, 2018 ▸). Model building and refinement were performed with *phenix.refine* (Adams *et al.*, 2010 ▸) with iterative rebuilding in *Coot* (Emsley *et al.*, 2010 ▸). Data-collection and refinement statistics are summarized in Table 1 ▸. Coordinates/structure factors have been deposited in the PDB with accession code 8bpd.

Molecular docking of Mef1 with a hexameric fucoidan model molecule as a ligand (the hexamer harbored the tetrameric product molecule resulting from Mef1-catalyzed hydrolysis) was accomplished using *CB-Dock* (https://cadd.labshare.cn/cb-dock2/; Liu *et al.*, 2022 ▸). Docking was initiated via the template-fitting procedure (Yang *et al.*, 2022 ▸); the hexameric fucoidan model molecule was placed in the scattered difference density in the active-site groove of Mef1 manually to align the sulfate groups in fucoidan with the presented CAPSO and ethylene glycol (EG) molecules; a structural outline of CAPSO is given in Supplementary Fig. S1.

All molecular graphics were prepared using *PyMOL* (version 2.2r7pre; Schrödinger).

## 3. Results and discussion

### 3.1. Discovery and sequence analysis of the Mef1 fucoidanase from *M. eckloniae*

The marine bacterium *M. eckloniae* strain DOKDO 007T belongs to the Flavobacteriaceae family; *M. eckloniae* was originally isolated from the rhizosphere of the brown alga *Ecklonia kuromea* and sequenced (accession No. PRJNA282771; Bae *et al.*, 2007 ▸). Mef1 was identified in the genome by the *BLAST* search engine (https://blast.ncbi.nlm.nih.gov/Blast.cgi) using selected and verified GH107 fucoidanases. Mef1 was found to be a putative GH107 fucoidanase of 399 amino acids in length including a 19-amino-acid predicted N-terminal signal peptide as indicated by *SignalP* 6.0 (Teufel *et al.*, 2022 ▸; https://services.healthtech.dtu.dk/service.php?SignalP-6.0).

**Figure 2:**
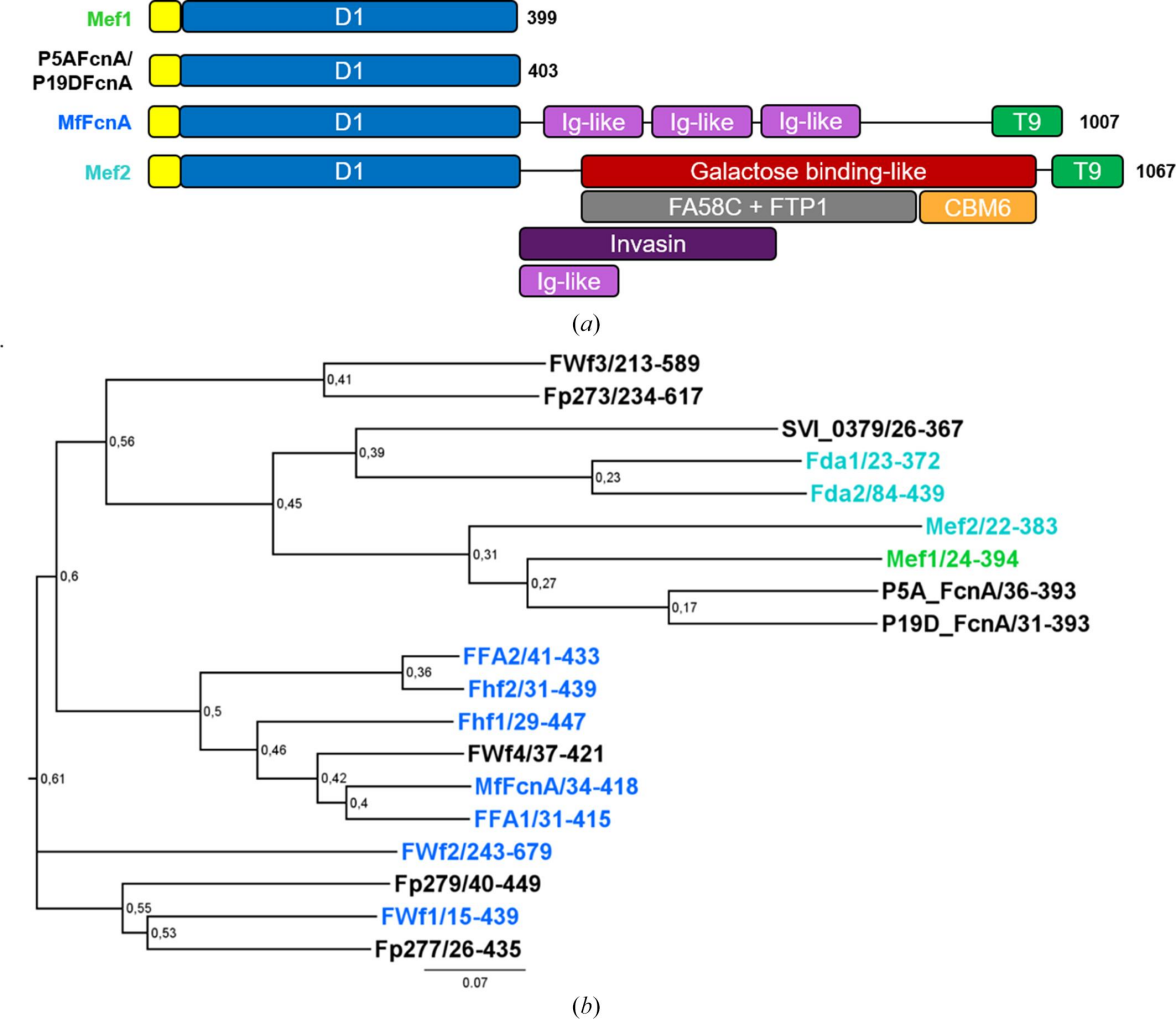
Domain prediction and phylogenetic analysis of Mef1. (*a*) Domain predictions of selected fucoidanases: Mef1, P5AFcnA/P19DFcnA, MfFcnA and Mef2. Yellow, signal peptide; blue, D1 (β/α)_8_ GH107 catalytic domain; pink, Ig-like domain (IPR003343); green, secretion-system C-terminal sorting domain (T9SS domain, T9; IPR026444); red, galactose binding-like domain (IPR000421); gray, FA58C domain (IPR000421) and FTP1 domain (IPR006585); orange, CBM6 domain (IPR005084); purple, invasin/intimin cell-adhesion domains (IPR008964). Domains were predicted using *SignalP* (signal peptide), *InterProScan* and sequence alignment (D1) with P5AFcnA and MfFcnA. (*b*) Phylogenetic analysis of the D1 domain amino-acid sequences of selected GH107 fucoidanases to help display how Mef1 is separated from other GH107 enzymes (enzyme codes and the microbial origin of each enzyme are given in Section 2.3); the numbers after each enzyme code denote the sequence frame used in the analysis for each protein. The phylogenetic tree was constructed with maximum likelihood using *phyML* and supported by likelihood-ratio tests resulting in values from 0 to 1, comparable to bootstrap values. The scale bar at the bottom and the number 0.07 is an indicator of the genetic distance based on branch length. Turquoise, α(1,3)-linkage-specific fucoidanases; green, Mef1; blue, α(1,4)-linkage-specific fucoidanases.

The catalytic domain (D1) was predicted in Mef1 based on sequence alignment with MfFcnA (Vickers *et al.*, 2018 ▸). Mef1 was found to be a single-domain protein, like P5AFcnA, only encompassing the D1 catalytic domain (Fig. 2 ▸ *a*). D1 of Mef1 shows a broad sequence-identity span ranging from 17% to 51% when compared with other characterized GH107 fucoid­anases (Supplementary Table S4). P5AFcnA (51%) and P19DFcnA (49%) had the highest sequence identity, followed by Mef2 (30%), Fda2 (22.9%) and Fda1 (21.5%) (Supplementary Table S4 and Fig. S2).

Sequence alignment (Supplementary Fig. S2) and phylogenetic analysis based on the D1 catalytic domain sequences revealed Mef1 to be grouped with Mef2, P5AFcnA and P19AFcnA (Fig. 2 ▸ *b*).

Due to the limited number of deposited sequences of family GH107 endo-fucoidanases, the phylogenetic analysis was only used to provide sequence-alignment similarity estimations and does not allow firm conclusions to be drawn regarding possible subfamily clusters of the enzymes.

The catalytic site residues of GH107 fucoidanases have been predicted to include a conserved histidine (His276 in P5AFcnA) and an aspartate (Asp201 in P5AFcnA) (Table 2 ▸) that act as the acid/base catalyst and the likely nucleophile, respectively (Vickers *et al.*, 2018 ▸). The histidine and aspartate catalytic amino acids were identified by sequence alignment as His270 and Asp187, respectively, in Mef1 (Table 2 ▸, Supplementary Fig. S2). In addition, three of the four previously suggested amino acids in the −1 subsite (Vickers *et al.*, 2018 ▸) were identified as Tyr128, Asn215 and Trp318 in Mef1 (Table 3 ▸, Supplementary Fig. S2).

The fourth amino acid in the −1 subsite was however not conserved in Mef1, as Asn145 in P5AFcnA was an alanine (Ala130) in Mef1 (Table 3 ▸). Neither was this residue conserved among the aligned fucoidanases: it was also an alanine in Fda1 and Fda2 and was a serine in Mef2 (Table 3 ▸). However, a proline, Pro313 in Mef1, was conserved in all of the aligned fucoidanases (Table 3 ▸, Supplementary Fig. S2), suggesting an important role of this amino acid (Tables 2 ▸ and 3 ▸, Supplementary Fig. S2).

### 3.2. Expression, purification and crystallization of Mef1

SDS–PAGE of the purified Mef1 showed a band with the expected molecular weight of 45 kDa; the presence of Mef1 was verified by Western blot analysis using anti-His antibodies (Supplementary Fig. S3). The initial affinity purification yielded Mef1 with greater than 95% purity; this was followed by size-exclusion chromatography and the data corroborated that the native Mef1 protein was a monodisperse monomer in solution with an expected size of approximately 42 kDa, resulting in a single protein band on SDS–PAGE with a purity above 99% (Supplementary Fig. S4).

Optimal crystals formed in hanging drops with 24% PEG 3350, 0.1 *M* CAPSO pH 9, 3% 2-propanol, 4% PEG 400. The crystals formed after ∼3 days and were subsequently picked, cooled and subjected to X-ray diffraction studies to determine the structure. Attempts to co-crystallize the crystals with fucoidan substrate and/or products were unsuccessful.

### 3.3. Crystal structure analysis of Mef1

**Figure 3:**
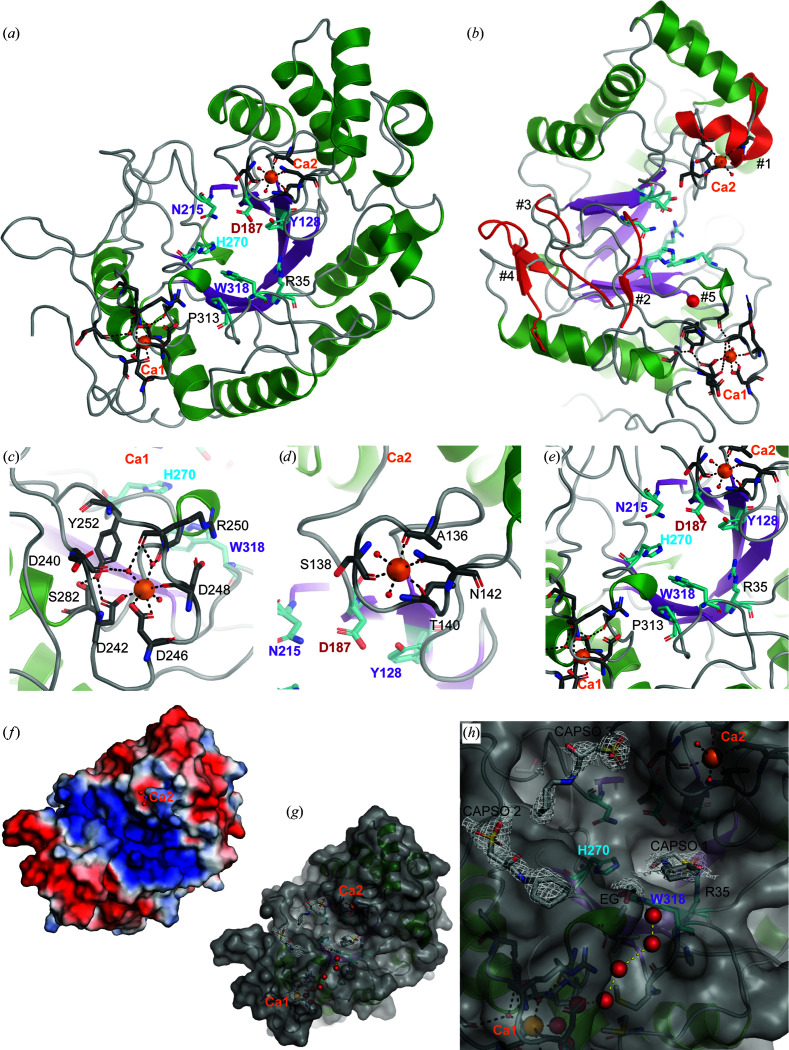
Crystal structure of Mef1. (*a*) The monomeric crystal structure of Mef1 (PDB entry 8bpd), illustrated as a cartoon, showing Ca^2+^-binding sites (Ca1 and Ca2) as well as the active site in cyan stick representation and with amino acids indicated with numbered single-letter codes (the active-site H270 is indicated in blue and the active-site aspartate D187 in red, while the −1 subsite amino acids are depicted in purple); the active-site β-barrel is indicated in purple, α-helices in green, random coils in gray and Ca^2+^ ions as orange spheres. (*b*) Superimposition of the crystal structures of the Mef1 and P5AFcnA enzymes. Deviations between the two crystal structures are shown in cartoon representation, with the secondary structure in P5AFcnA presented in red (#1–#5). The Ca^2+^ sites are highlighted in (*c*) for Ca1 and (*d*) for Ca2, where the Ca^2+^-coordinating amino acids are indicated as sticks with numbered single-letter codes. (*e*) Magnification of the active site, highlighting the amino acids lining the β-barrel region of Mef1 (purple) in (*a*). (*f*) Surface-charge representation of the Mef1 crystal structure (blue, positively charged amino acids; red, negatively charged amino acids; Ca2 is indicated as a purple ball, with two coordinating O atoms as red balls). (*g*) Mef1 crystal structure including the modeled CAPSO and EG molecules and the water wire (red spheres). (*h*) Magnification of the active site of Mef1 in (*g*), showing the CAPSO and EG molecules as well as the water wire (red spheres).

The 1.8 Å resolution crystal structure (Table 1 ▸) of Mef1 revealed a (β/α)_8_-barrel structure with a similar structural architecture to that observed for the homologous D1 domain of the GH107 endo-fucoidanases P5AFcnA (PDB entry 6m8n) and MfFcnA (PDB entry 6dlh) (Vickers *et al.*, 2018 ▸; Fig. 3 ▸ *a*). The active site of Mef1 was situated at the center of the β-barrel and included the catalytic amino acids His270 and Asp187 and the three conserved amino acids of the −1 subsite Tyr128, Asn215 and Trp318 (Fig. 3 ▸ *a*); numbering of the sugar-binding subsites was performed according to the classical nomenclature originally proposed for endo-acting glycoside hydrolases (Davies *et al.*, 1997 ▸).

A proline conserved in GH107, Pro313 in Mef1, was found near the active site, where it forms a CH–π interaction with Trp318 and Pro313 in the −1 subsite and is therefore likely to be important for the proper positioning of Trp318 in the −1 subsite (Fig. 3 ▸ *a*). The active site is located in the center of an elongated groove presented on the surface of Mef1. The amino acids in the groove are largely positively charged, as is especially evident for Arg35, and are likely to assist with the binding and positioning of the largely negatively charged sulfated fucoidan polysaccharide. Indeed, Arg35 appears to be involved in the binding of the sulfate group of CAPSO by Mef1 (Fig. 3*h*; discussed further below).

#### 3.3.1. Calcium sites in the Mef1 structure

Densities corresponding to two Ca^2+^ ions were identified in the crystal structure of Mef1 (Fig. 3 ▸): a Ca^2+^ ion (Ca1) was found in a very well organized Ca^2+^ site coordinated by Asp242, Asp246 and Asp248 (Table 4 ▸) and by an H_2_O bound to Asp240 through hydrogen bonds (Fig. 3 ▸ *c*). The Ca1 site is further stabilized by hydrogen bonds from Tyr252 to Asp242 and from Ser282 to Tyr252 (Fig. 3 ▸ *c*). The Ca1 site thus seems to be very tightly coordinated; interestingly, the backbone of Arg250 is likely to be involved in bifurcated hydrogen bonding to two ligand water molecules, which coordinate Ca1, while the carboxyl group coordinates via hydrogen bonds to Asp248. A possible additional Ca^2+^-binding site (Ca2) was found close to the active-site residue Asp187 (Fig. 3 ▸ *d*).

The coordination of Ca2 was mediated by the backbone carbonyl groups of Ala136, Ser138 and Thr140 and the amide group of Asn142 (Fig. 3 ▸ *d* and Table 4 ▸); the Ca2 site lines the active-site groove and has two coordinating water molecules. The geometry of this site would also allow a magnesium ion to bind, and it therefore cannot be ruled out that another similar ion could occupy this site under natural conditions in the ocean. However, in this structure it is a calcium ion as dictated by the crystallization conditions. Both CAPSO and ethylene glycol (EG) were present during crystallization, which revealed four additional difference density regions in Mef1. One possible EG is located in the active site, along with three possible CAPSO molecules: one located in the active site (CAPSO 1) and two on the surface of Mef1 (CAPSO 2 and 3) (Figs. 3 ▸ *f* and 3 ▸ *g*). CAPSO, which is normally used as a buffer, is a small, sulfated molecule and the sulfate group of CAPSO 1 is coordinated in the Mef1 crystal primarily through Arg35, while the EG site is close to a small α-helical structure stabilized by Ca1. The latter is likely to act as a dipolar charge combined with coordination from both active-site amino acids His270 and Trp318 to stabilize the EG.

The possible sulfate site, coordinated by Arg35 (CAPSO 1), is not conserved in the sequence alignment with P5AFcnA. In the sequence alignment, Arg53 was present two residues away from the equivalent site in Mef1 (Supplementary Fig. S2). However, in the P5AFcnA structure (PDB entry 6m8n) Arg53 is in a different position further away from the lining of the central active-site β-barrel than Arg35 in Mef1. Although we did not find conservation in the sequence alignment in other GH107 fucoidanases, structural conservation is possible with arginine present in this other position. Indeed, there is sequence conservation in this position in some of the other investigated GH107 fucoidanases in an alignment of P5AFcnA, P19AFcnA, Fda1, Fda2 and Svi_0379 (Supplementary Fig. S2). While the primary structures of Mef1 and P5AFcnA (PDB entry 6m8n) share a sequence identity of only 53%, the crystal structures share strong structural similarity, with an overall r.m.s.d. of 0.7 Å when superimposed. The major deviations between the two crystal structures were mainly found in the loop regions towards the putative +1 subsite. The deviations identified are numbered #1–#5 and are visualized in Fig. 3 ▸(*b*). Deviation #1 from residues Ser135 to Asp145 forms a coil that coordinates a Ca^2+^ site in Mef1, while in P5AFcnA this region forms a short α-helical structure that is unable to coordinate any divalent metal ions. The second deviation (#2) from Asn215 to Tyr228 in Mef1 is close to the active site and forms a coiled structure in both Mef1 and P5AFcnA; however, it is extended in Mef1.

A similarly extended coil is present in Mef1 at the third deviation (#3) extending from Ser256 to Tyr263. P5AFcnA presents an additional two-sheet β-sheet structure at the fourth deviation, while residues Asp295–Lys308 in Mef1 form a shortened random-coil structure in Mef1. The site of the fifth deviation (#5) indicates the presence of a Ca^2+^ site in P5AFcnA, which was positioned differently compared with the two Ca^2+^ sites found in Mef1. The Ca^2+^ site identified in P5AFcnA was subjected to closer inspection using the map coefficients supplied for PDB entry 6m8n; the coordination and ligand environment would make it more likely that a chloride ion should be present at this position. This analysis also revealed that a sodium ion is likely to occupy a site equivalent to the Ca1 site in Mef1, thus underscoring the importance of a metal ion in this position.

While the alignments of the two Mef1 Ca^2+^ sites revealed poor conservation compared with other described GH107 members, the Ca1 site is almost conserved in P19AFcnA except for Asp240 being an alanine and being completely conserved in P5AFcnA (Table 4 ▸). From investigations of the crystal structure of P5AFcnA (PDB entry 6m8n) it was evident that density was present in this location, which could be a sodium molecule. The crystallization conditions for P5AFcnA did not contain Ca^2+^ (Vickers *et al.*, 2018 ▸) and this site could therefore be a comparable Ca^2+^ site to that found in Mef1. Asp252 in P5AFcnA (corresponding to Asp246 in Mef1) was however refined as an alanine in the crystal structure, although written correctly as an aspartic acid. When properly refined, we found structural conservation in the Ca1 coordination in P5AFcnA and Mef1.

The Ca^2+^ site previously predicted in the P5AFcnA crystal structure was described as weakly bound by an N atom of Lys259 and a water molecule (Vickers *et al.*, 2018 ▸), but the density in this location and the coordination is very convincingly a Cl^−^ ion and not a Ca^2+^ ion.

The very tight coordination of Ca1 in Mef1, leaving one coordination site free, with the coordination of the other sites being extremely tight, suggests an important role of this calcium site in Mef1. The Ca1 site is not near the active site, which could indicate a role other than direct catalytic involve­ment.

A water wire was identified leading from the exterior of the Mef1 protein, closely passing the free coordination site of Ca1 and further passing directly into the active site (Figs. 3 ▸ *f* and 3 ▸ *g*). This water wire is lined by a hydrophilic and a hydrophobic stretch of amino-acid residues stretching from Ca1 to the EG molecule, which is in contact with the active-site residues Trp318 and His270 (Fig. 3 ▸ *g*). This water wire might be involved in carrying the excess proton from the hydrolytic catalysis of fucoidan.

The possible CAPSO molecule in the active site of Mef1 could be important for the crystallization of Mef1, since Mef1 crystals could only be obtained when adding CAPSO, or a small analog called CHES, to the crystallization mixture. CAPSO and CHES were investigated for possible inhibitory effects on the fucoidanase activity of Mef1 by C-PAGE analysis. Interestingly, increasing concentrations of both molecules showed an inhibitory effect on Mef1 activity (Supplementary Fig. S5). At concentrations of over 250 and 600 m*M* for CAPSO and CHES, respectively, the Mef1 enzyme was completely inactive. It could be speculated that CAPSO and CHES could bind to the active site considering that their sulfonic acid groups are analogous to the sulfate substitutions on fucoidan. This hypothesis is consistent with the CAPSO found blocking the active site in the crystal, leaving the fucoidanase inactive and structurally inflexible while enabling crystallization. The optimized Mef1 crystals in CHES and CAPSO showed different crystal types (Supplementary Figs. S4*c*–4*e*) and formed at different times; with CAPSO the crystals formed after 3–4 days, while with CHES they formed later after around 7–10 days. Investigating the effect of these two buffers on Mef1 enzyme activity resulted in inhibition of the Mef1 enzyme. After 24 h incubation with CHES, the enzyme was inactivated at 250 m*M* concentration, while the enzyme was inactivated after 30 min of incubation at 250 m*M* CAPSO.

### 3.4. Substrate specificity and action of Mef1

**Figure 4:**
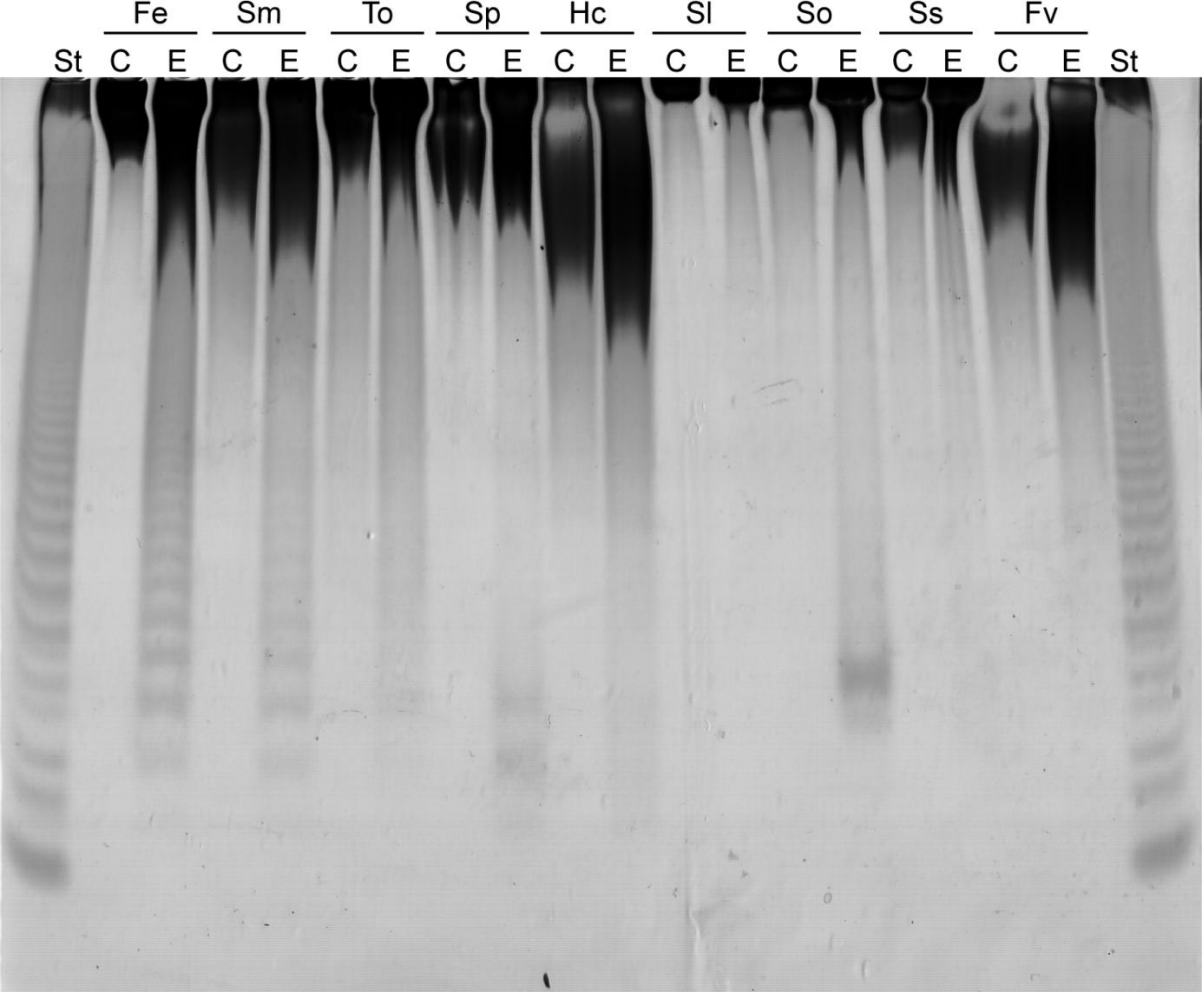
Substrate specificity of Mef1 on different brown seaweed fucoidans by carbohydrate polyacrylamide gel electrophoresis (C-PAGE) after 2 h of reaction. Lanes C, fucoidan substrate controls for each type of fucoidan; lanes E, enzyme action of Mef1 on the fucoidans from *F. evanescens* (Fe), *S. mcclurei* (Sm), *T. ornata* (To), *S. polycystum* (Sp), *H. cuneiformis* (Hc), *S. latissima* (Sl), *S. oligocystum* (So), *S. serratum* (Ss) and *F. vesiculosus* (Fv). St designates the hydrolysate standard obtained after the enzymatic reaction of FFA2 [an α(1,4)-linkage-cleaving GH107 fucoidanase] on *F. evanescens* fucoidan with all fucose residues sulfated at C2; the lowest band is a tetrasaccharide (Silchenko *et al.*, 2017 ▸).

The substrate specificity of Mef1 was investigated by assessing the C-PAGE response on different fucoidans extracted from different species of brown seaweed. Fucoidan from *F. evanescens* containing alternating α(1,4)- and α(1,3)-glycoside bonds was efficiently depolymerized by the Mef1 enzyme (Fig. 4 ▸). However, Mef1 did not catalyze any significant hydrolysis of fucoidan from *S. latissima*, which mainly contains α(1,3)-glycosidic bonds (Bilan *et al.*, 2010 ▸), thus suggesting α(1,4)-bond selectivity. The fucoidan originating from *S. mcclurei* was also degraded by Mef1 (Fig. 4 ▸). The *S. mcclurei* fucoidan structure is very complex and consists of α(1,3)- and α(1,4)-linked fucosyl residues which are differentially sulfated at C2 and/or at C4, as well as α(1,4)- and α(1,6)-linked galactosyls (Thinh *et al.*, 2013 ▸; Fig. 1 ▸). Likewise, Mef1 showed activity on the two galactofucans from *S. oligocystum* and *S. polycystum*, both of which have been predicted to be branched fucoidans.

A time-course experiment of the hydrolysis of fucoidan from *F. evanescens* by Mef1 resulted in the accumulation of polydisperse oligomeric products as the reaction time increased, supporting an endo-acting enzyme activity (Supplementary Fig. S6*a*; exo-acting enzymatic hydrolysis will produce single monomeric products). According to the HP-SEC/RI analysis, the average molar mass of fucoidan-derived products decreased over time from approximately 650 to 8 kDa, corroborating that Mef1 is an endo-acting enzyme (Supplementary Fig. S6*b*).

#### 3.4.1. Product-formation analysis of the action of Mef1

**Figure 5:**
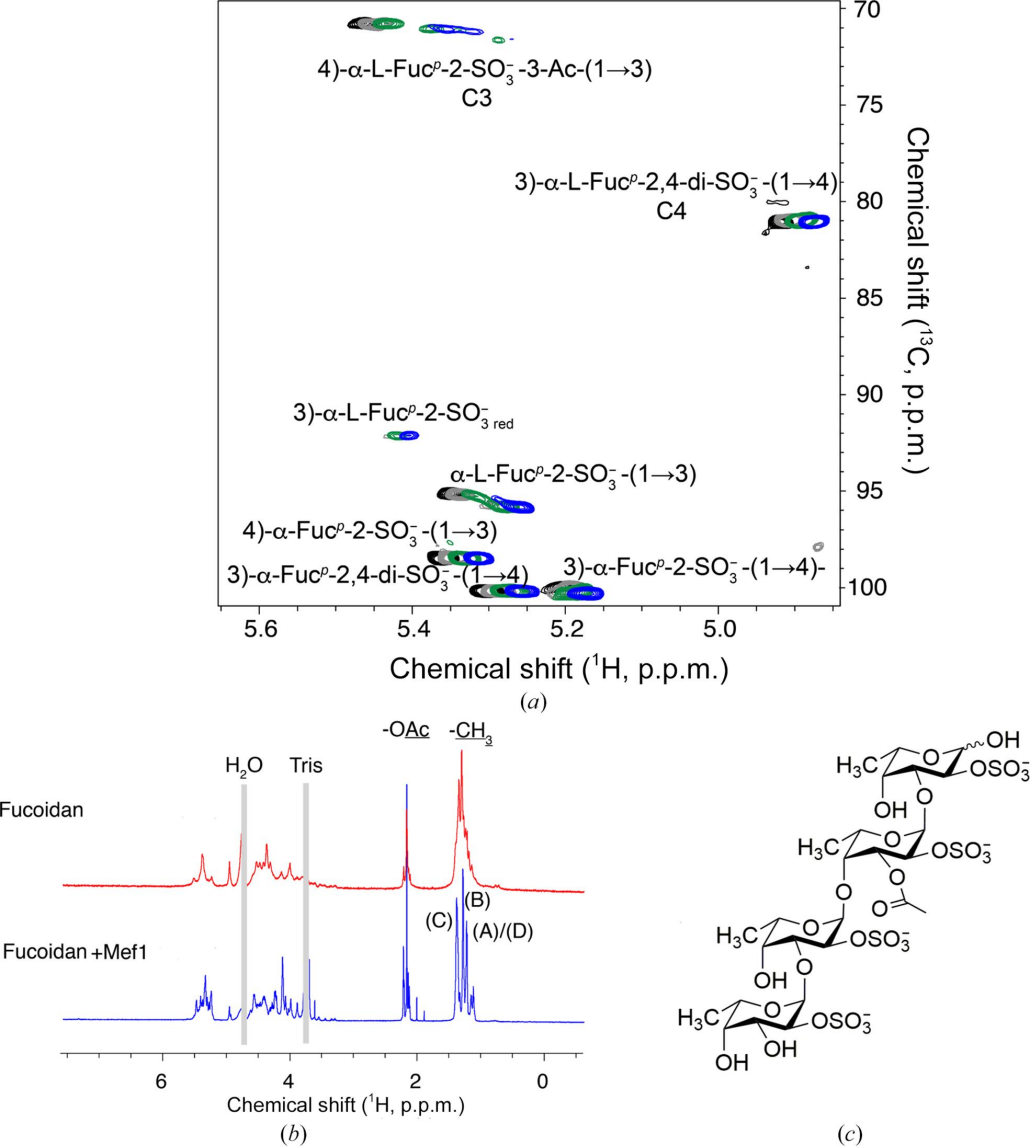
Product formation from Mef1 hydrolysis of fucoidan from *F. evanescens* as monitored by NMR spectroscopy. (*a*) *In situ* ^1^H–^13^C NMR spectroscopy of the degradation of fucoidan after 20 min (black), 120 min (gray), 400 min (green) and 800 min (blue) at 303 K and pH 8.0. The emergence of reducing-end signals and other transitions from substrate to product signals are due to enzyme activity. Spectra were manually offset in the ^1^H dimension by −0.02 p.p.m. between the time points for clarity, showing that glycosidic bonds at the anomeric C atom of 3)-α-Fuc*p*-2-SO_3_^−^-(1,4) residues are broken, while structural motifs containing 3)-α-Fuc*p*-2,4-di-SO_3_^−^-(1,4)-α-Fuc-2-SO_3_^−^-(1,3) repeating units remain intact. (*b*) ^1^H NMR spectra of *F. evanescens* fucoidan (fucoidan) and the low-molecular-weight product fraction upon degradation with Mef1 (fucoidan + Mef1). (*c*) Molecular structure of the tetrasaccharide constituting the principal low-molecular-weight hydrolysis product (LMP) upon degradation of *F. evanescens* fucoidan by Mef1.

The products of the action of Mef1 on *F. evanescens* fucoidan were verified in a real-time *in situ* tracking assay using ^1^H–^13^C NMR spectroscopy (Fig. 5 ▸). The anomeric region and acetyl­ation site in particular of *F. evanescens* fucosyl residues in different structural motifs were used to track time-dependent structural changes occurring in the ongoing reaction (Fig. 5 ▸ *a*). A lack of change in the signals indicated that the structural motifs were far from the cleavage site.

In Mef1 cleavage of *F. evanescens* fucoidan, the emergence of reducing-end signals with single sulfation at the C2 position and glycosidic linkages to the C3 position was paralleled by changes to structural motifs containing 3)-α-Fuc*p*-2-SO_3_^−^-(1,4)-α-Fuc-2-SO_3_^−^-(1,3), indicating that the α(1,4)-glycosidic bonds in this motif, which was also partly acetylated, were hydrolyzed. In contrast, structural motifs containing 3)-α-Fuc*p*-2,4-di-SO_3_^−^-(1,4)-α-Fuc-2-SO_3_^−^-(1,3) repeating units remained intact (Fig. 5 ▸ *a*).

To determine the linkage and sulfate specificity of Mef1, the degradation products were isolated from *F. evanescens* fucoidan after Mef1 hydrolysis. The low-molecular-weight hydrolysis products (LMP) were separated from the medium-molecular-weight products (MMP) by precipitation with 75% aqueous ethanol and were analyzed by recording a suite of NMR spectra including ^1^H–^1^H COSY, ^1^H–^1^H TOCSY, ^1^H–^13^C HSQC, ^1^H–^13^C HMBC and ^1^H–^1^H ROESY (Fig. 5 ▸). Since a second Mef1 enzyme-assay step did not result in additional release of LMP, we conclude that all cleavable sites in fucoidan specific to Mef1 had been depleted during the first reaction on fucoidan from *F. evanescens*.

The ^1^H NMR spectrum of the Mef1 LMP was different from the native *F. evanescens* fucoidan, particularly in the regions of the α-anomeric protons (5.6–5.3 p.p.m.) and the H2 and H4 protons of the sulfate groups, as well as fucose methyl groups (1.24–1.32 and 1.38–1.41 p.p.m.; Fig. 5 ▸ *b*, Supplementary Table S6). Mef1-derived fragments of *F. evanescens* fucoidan were analyzed by 2D NMR spectroscopy. The signals within each spin system were assigned primarily based upon ^1^H–^1^H COSY, ^1^H–^1^H TOCSY, ^1^H–^13^C HMBC and ^1^H–^13^C HSQC correlations.

Linkage analyses of the oligosaccharide were performed using ^1^H–^13^C HMBC experiments to detect ^3^ *J*_CH_ correlations across the glycosidic bond, and were aided by comparison to ample literature data relating to NMR data of fucoidan fractions (Bilan *et al.*, 2002 ▸, 2004 ▸). Fragments were found to encompass tetrasaccharides resulting from the cleavage of α(1,4)-glycosidic bonds. The reducing-end unit (*i.e.* the −1 position at the cleavage site) was found to be monosulfated at C2, as shown by a characteristic H2 chemical shift, and linked to the next fucose unit at the C3 position: the −2 position at the cleavage site. This second fucose unit was predominantly acetylated at the C3 position and was sulfated at the C2 position. In contrast, the nonreducing-end unit was primarily non-acetylated (Fig. 5 ▸ *c*). The Mef1 enzyme thus seems to selectively catalyze the cleavage of α(1,4)-glycosidic linkages between non-acetylated and monosulfated units in structural motifs that contain mono-acetylated units at the −2 position. Sulfation at C2 was observed at each fucose unit from the chemical shift values for H2 of the order of 4.5–4.7 p.p.m., whereas desulfated residues would exhibit chemical shift values in the range 3.8–4.0 p.p.m. for H2. Similarly, acetylation at C3 of residue C in the tetrasaccharide product was observed from the characteristically high ^1^H chemical shift at the H3 position of 5.39 p.p.m.. The molecular structure of the tetrasaccharide (including an acetyl group) constituting the principal LMP upon the degradation of *F. evanescens* fucoidan with Mef1 was thus identified as a C2-sulfated fucose tetrasaccharide with the cleavage site of fucose −2 acetylated on C3 (Fig. 5 ▸ *c*). The units have the same identifiers as in Supplementary Table S3.

The scarcity of 2,4-disulfation in the tetrasaccharide was deducted from the weaker intensity of a characteristic H4 signal (with a ^1^H chemical shift near 4.95 p.p.m.), which is strong in the intact fucoidan and in the MMP containing -3)-α-Fuc*p*-2,4-di-SO_3_^−^-(1,4)-α-Fuc-2-SO_3_^−^-(1- repeating units. These repeating units are thus not affected by the enzymatic degradation of *F. evanescens* fucoidan with Mef1 and constitute a medium-molecular-weight byproduct of the enzymatic cleavage. The same fragments were formed upon degradation by Mef1 and Fhf1, as shown by the highly similar ^1^H–^13^C NMR spectra of the LMP upon enzyme treatment with either enzyme (Supplementary Fig. S7). The spectra showed that the MMP fraction contains few acetylated units compared with the substrate, and the MMP is apparently predominantly comprised of -3)-α-Fuc*p*-2,4-di-SO_3_^−^-(1,4)-α-Fuc-2-SO_3_^−^-(1- repeating units, while the LMP fraction contains partially acetylated, monosulfated fragments (Supplementary Fig. S8). Previous results have shown similar MMP products to be formed by Fhf1 on deacetylated fucoidan from *F. evanescens* (Vuillemin *et al.*, 2020 ▸). The current results were obtained with native and acetylated fucoidans, indicating that both Fhf1 and Mef1 prefer acetylation at C3 in the active-site −2 position.

Overall, Mef1 is thus suggested to specifically catalyze the cleavage of α(1,4)-linkages between fucosyl residues sulfated on C2 in the structural motif -3)-α-l-Fuc*p*2S-(1,4)-α-l-Fuc*p*2S-(1-. Similar to Mef1, Mef2 has been found to cleave *F. evanescens* fucoidan in structural motifs that do not contain 2,4-disulfated fucoidan residues and to liberate fragments with 1–3-linked and 1–4-linked alternating monosulfated units (Tran, Nguyen *et al.*, 2022 ▸). In contrast to Mef1, Mef2 has been characterized as an endo-α(1,3)-fucoidanase. Cleavage of monosulfated stretches still leaves a similar MMP fraction for Mef1 and for Mef2 cleavage of *F. evanescens* fucoidan that contains intact domains with 2,4-disulfated fucosyl units and without significant acetylation (Supplementary Figs. S7 and S9).

### 3.5. Molecular docking

**Figure 6:**
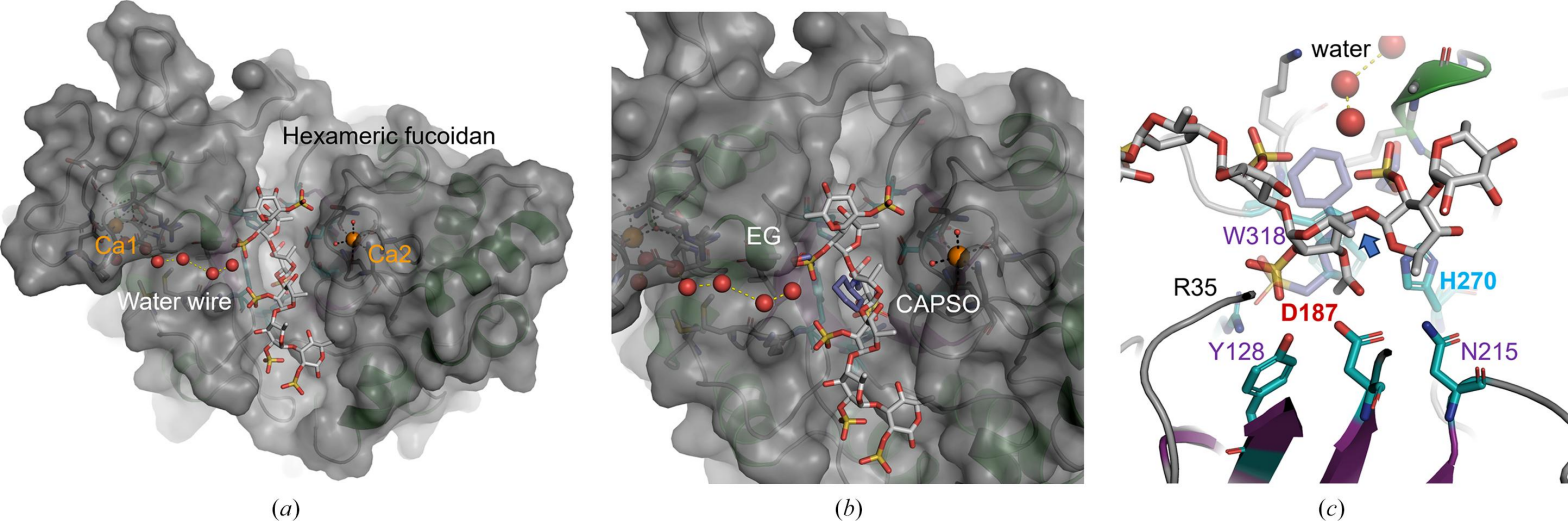
Molecular docking showing the binding mode of Mef1 and a hexameric fucoidan molecule (harboring the tetrasaccharide product). (*a*) Structural model of the Mef1 surface with the hexameric fucoidan ligand shown as a stick model; the water wire (red spheres) and calcium ions (orange spheres) are also shown. (*b*) A close-up of the CAPSO (transparent blue) and the ethylene glycol (EG; transparent blue) positions; after docking, the CAPSO (equivalent to CAPSO 1 in Fig. 3 ▸ *h*) and EG remain superimposed with the hexameric fucoidan molecule. (*c*) Magnification of a stick model of the active-site residues in close proximity to the fucoidan hexamer, with the scissile α(1,4)-bond indicated by a blue arrow. The CAPSO and EG positions are presented as transparent blue sticks.

Molecular docking of a hexameric C2-sulfated fucoidan containing the tetrameric *F. evanescens* fucoidan product into the active site of Mef1 displayed how well the active site accommodates the fucoidan ligand (Fig. 6 ▸ *a*). Indeed, the docking data also showed complete congruence between the positioning of one of the fucoidan sulfate groups and one of the sulfonates of a CAPSO molecule (Figs. 6 ▸ *b* and 6 ▸ *c*), which is in full agreement with the crystallization data showing the CAPSO 1 and ethylene glycol positions (Fig. 3 ▸ *h*). Thus, the positioning of the sulfate groups of the fucoidan substrate is likely to contribute to the substrate selectivity and the formation of the reactive Mef1 enzyme–substrate complex. The amino acids lining the active-site cleft, including Arg35, are likely to support the correct substrate positioning. During catalysis, the sulfated fucoidan is thus expected to approach the active-site residues to allow nucleophilic attack and the catalytic cleavage of an α(1,4) bond via His270 and Asp187 (Fig. 6 ▸ *c*).

### 3.6. Divalent cation dependence, pH and temperature optimum of Mef1

Mef1 was found to be active in a wide pH range spanning pH 6.0 to 10.0 (Supplementary Fig. S9). Within the pH range 7.0–9.0 all LMPs were visible after 2 h of reaction time (Supplementary Fig. S9*a*). The optimal pH of Mef1 was around pH 8.0, where all LMPs were released in clearly visible amounts compared with the other pH values (Supplementary Figs. S10*a* and S10*b*). In addition, the effect of different buffers was investigated, including Tris–HCl, borate, sodium phosphate and UB4 buffer at pH 8.0. These results confirmed that both Tris–HCl and UB4 buffers are suitable for Mef1 enzyme-activity assays (Supplementary Fig. S9*c*).

Mef1 showed activity at NaCl levels ranging from 25 to 400 m*M*, with an optimum in the range 50–150 m*M*, while almost complete inactivation was observed at 500 m*M* NaCl (Supplementary Fig. S10*a*). The enzyme was subjected to EDTA treatment and desalting prior to the investigation of the influence of different divalent cations (Ca^2+^, Mg^2+^, Mn^2+^, Cu^2+^, Fe^2+^, Zn^2+^, Co^2+^ and Ni^2+^; Supplementary Fig. S10*b*). Treatment with EDTA reduced the enzymatic activity as assessed by C-PAGE (Supplementary Fig. S10*b*). It was not possible to restore the activity of Mef1 by addition of the divalent cations Cu^2+^, Fe^2+^, Zn^2+^, Co^2+^and Ni^2+^, while the activity was restored by the addition of Mg^2+^, Mn^2+^ and notably of Ca^2+^. Together with the Ca^2+^ sites found in the structural analysis of Mef1, this result validated that Mef1 is a Ca^2+^-dependent enzyme, equivalent to previously characterized GH107 fucoidanases (Vuillemin *et al.*, 2020 ▸; Trang *et al.*, 2022 ▸; Tran, Nguyen *et al.*, 2022 ▸; Silchenko *et al.*, 2017 ▸).

The optimal temperature range for Mef1 was estimated to be between 20 and 37°C (Supplementary Fig. S11*a*), with the highest production of visible smaller fucoidan oligosaccharides at 37°C (Supplementary Fig. S11*a*). As expected, the enzyme activity decreased at 40–45°C, and temperatures above 50°C inactivated the enzyme. These findings were supported by thermal stability assessment (Supplementary Fig. S11*b*) and determination of the melting temperature (*T*_m_) of Mef1, which was ∼43°C. When the divalent cations were removed by EDTA, the *T*_m_ was reduced to 40°C, indicating that divalent ions (likely Ca^2+^) have a stabilizing effect on the Mef1 structure; *T*_m_ increased further to 46°C in the presence of Ca^2+^ when the substrate was also present, corroborating stabilizing effects of both Ca^2+^ and substrate (Supplementary Table S5).

The optimal conditions for the activity of Mef1 on fucoidan from *S. oligocystum* were similar to those for that from *F. evanescens* (Supplementary Fig. S12). Although the production of oligosaccharides was considerably lower on *S. oligocystum* fucoidan than on *F. evanescens* fucoidan, the detection of activity on *S. oligocystum* fucoidan is noteworthy, since no fucoidanase to date has been found to be active on this substrate (Supplementary Fig. S12). Mef1 is thus the first enzyme demonstrated to catalyze the hydrolysis of fucoidan from *S. oligocystum*.

### 3.7. Mef1 kinetics by FTIR spectroscopy

**Figure 7:**
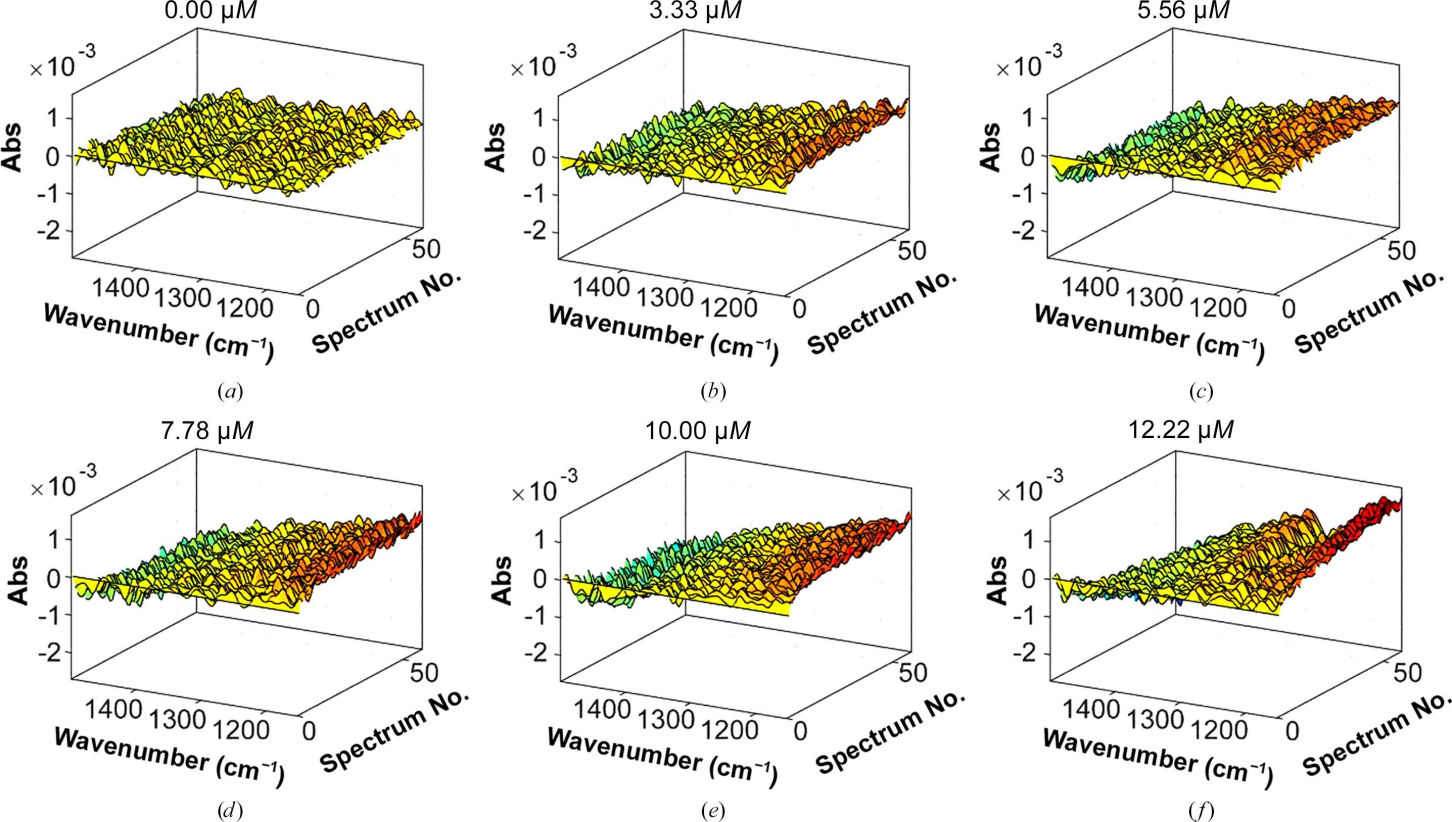
FTIR spectral evolution profiles for different dosages of Mef1 on 2%(*w*/*v*) fucoidan from *F. evanescens*. The spectral evolution depends on the enzyme concentration. The spectral changes from buffer and substrate were subtracted so that net spectral changes of enzyme activity were visualized.

The evolution of spectral changes with increasing concentrations of Mef1 revealed an increase in absorption at wavenumbers of 1400–1300 cm^−1^ and a decrease in absorption at wavenumbers of 1250–1150 and 1500 cm^−1^ (Fig. 7 ▸). The spectral changes observed at 1400–1300 cm^−1^ could be caused by stretching vibrations in the C—OH bonds, including the contribution of O—C—O symmetric stretching vibrations of the carboxylate group. Spectral changes at wavenumbers of 1250–1150 cm^−1^ are likely to represent the stretching vibrations in sulfate ester groups, thus showing spectral changes upon the degradation of sulfated fucoidan. Changes in these wavenumbers have previously been observed during the hydrolysis of fucoidans from *F. evanescens* by the fucoidanases MfFcnA, Fhf1, FFA2 (Tran, Perna *et al.*, 2022 ▸), Fhf2 (Trang *et al.*, 2022 ▸) and Mef2 (Tran, Nguyen *et al.*, 2022 ▸).

**Figure 8:**
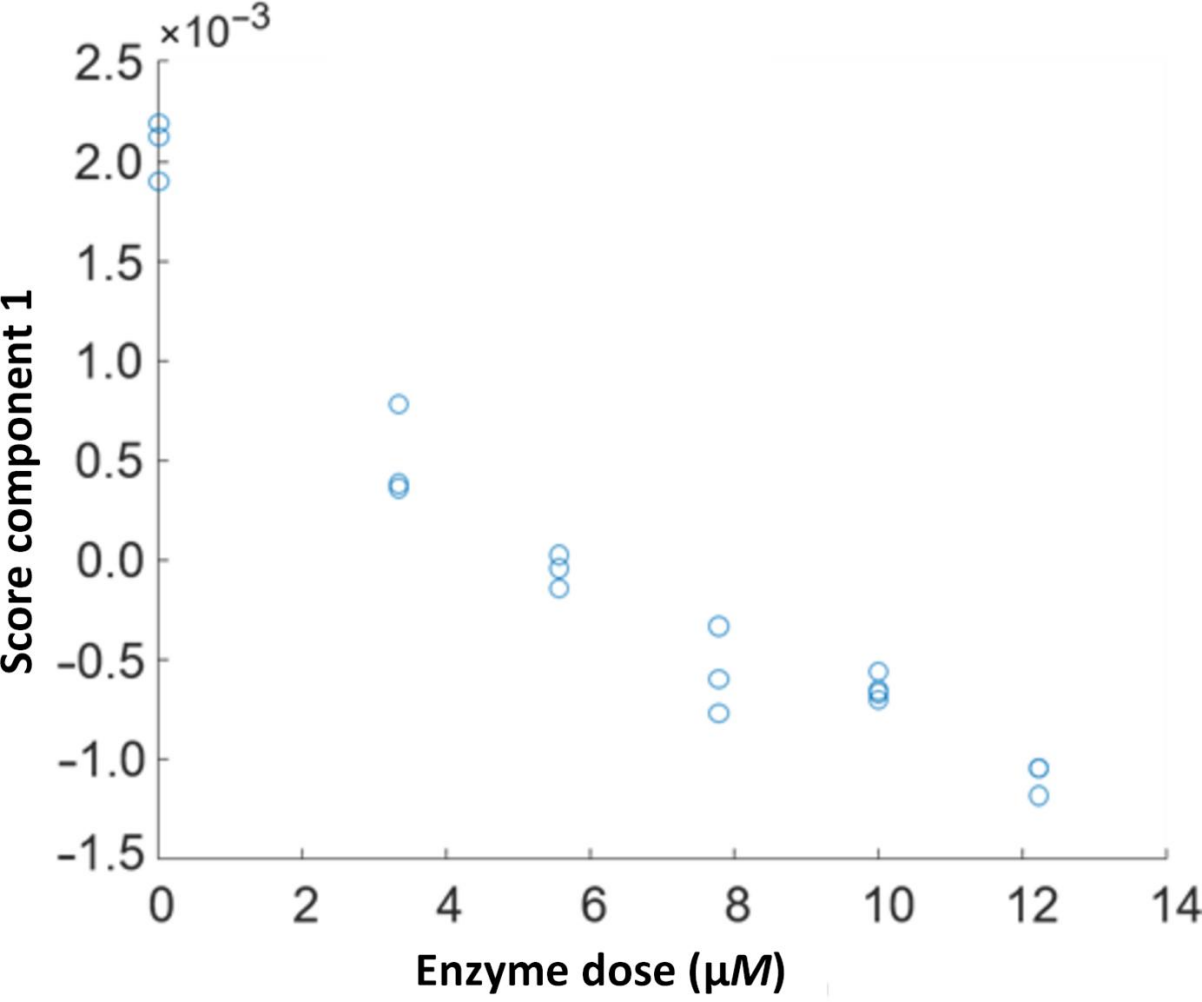
Mef1 FTIR data scores, interpreted using PARAFAC first-component scores versus enzyme dosage. Modeled linear regression slope: *y* = −0.0001[enzyme dose] + 0.0005, *R* ^2^ = 0.92.

The three-dimensional results of the FTIR spectroscopy of fucoidanase action (Fig. 7 ▸) can be converted into a linear curve in two dimensions by PARAFAC (Harshman & Lundy, 1994 ▸) to directly reflect the concentration-dependent enzyme kinetics. PARAFAC analysis of the concentration-dependent Mef1-catalyzed hydrolysis of fucoidan resulted in a linear plot with the equation *y* = −0.0001*x* + 0.0005 and an *R* ^2^ value of 0.92 (Fig. 8 ▸).

One enzymatic unit U_f_ is defined as the concentration of enzyme that is able to increase the value of the FTIR PARAFAC score by 0.01 (Tran, Perna *et al.*, 2022 ▸). For Mef1, the enzyme concentration equivalent to a numeric change in the score of 0.01 was

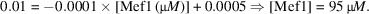

Hence, the specific activity of Mef1 was 0.1 × 10^−3^ U_f_ µ*M* ^−1^.

The Mef1 activity on *F. evanescens* was higher than the activity of Fhf2 (2.4 × 10^−4^ U_f_ µ*M* ^−1^; Trang *et al.*, 2022 ▸), but lower than the activities of other GH107 endo-fucoidanases characterized by this FTIR assay, for example MfFcnA (2.0 × 10^−3^ U_f_ µ*M* ^−1^), FFA2 (4.0 × 10^−3^ U_f_ µ*M* ^−1^) and Fhf1 (1.2 × 10^−3^ U_f_ µ*M* ^−1^), and about ten times lower than the activity of Mef2 (1.2 × 10^−3^ U_f_ µ*M* ^−1^; Tran, Nguyen *et al.*, 2022 ▸).

## 4. Discussion

In this study, we structurally and functionally characterized a new GH107 fucoidanase, Mef1, originating from the marine bacterium *M. eckloniae*. Mef1 and several other previously characterized fucoidanases, such as Fhf1, Fhf2, Fda1, Fda2, MfFcnA and FFA2 (Colin *et al.*, 2006 ▸; Vuillemin *et al.*, 2020 ▸; Trang *et al.*, 2022 ▸; Cao *et al.*, 2018 ▸; Silchenko *et al.*, 2017 ▸), contain an N-terminal signal peptide and a catalytic D1 domain. However, Mef1 deviates from the abovementioned fucoidanases by not containing an extended C-terminal domain and thus represents a minimal fucoidanase. The C-terminal domains in fucoidanases such as Fhf2 (Trang *et al.*, 2022 ▸), Fda1 and Fda2 (Cao *et al.*, 2018 ▸) have not been found to be crucial for activity in *in vitro* experiments; rather, C-terminal truncation stabilized the enzymes. The closest structural homologs of Mef1 include P5AFcnA and P19AFcnA (Vickers *et al.*, 2018 ▸), which also lack a C-terminal extension. The linkage specificities of the P5AFcnA and P19AFcnA fucoidanases remain undetermined, while the linkage specificity of Mef2 has been characterized to be α(1,3) (Tran, Nguyen *et al.*, 2022 ▸; Takayama *et al.*, 2007 ▸); it is interesting to note that Mef1 and Mef2 only have 30% sequence identity.

The present study establishes that Mef1 is an α(1,4)-specific fucoidanase rather than an α(1,3)-specific fucoidanase, which leads to the suggestion that phylogenetic relationships in the GH107 family have to be considered carefully when predicting the specificities of the members according to only sequence-based phylogeny while the sequence base is as small as it is at present.

The crystal structure of Mef1 shows a classical (β/α)_8_-barrel structure with an active site positioned in the β→α loops (Reardon & Farber, 1995 ▸), equivalent to the active site of P5AFcnA and MfFcnA described by Vickers *et al.* (2018 ▸). There are, however, significant differences between the structures. In particular, the structure of P5AFcnA (PDB entry 6m8n) only reports a single Ca^2+^-binding site, while Mef1 has two Ca^2+^-binding sites (Ca1 and Ca2). In Mef1, Ca1 is a highly coordinated site, with Asp242 and Asp246 contributing with their side-chain carboxyl groups, while Asp248, Arg250 and Asp240 contribute with their backbone carbonyl groups. Two water molecules complete the heptavalent co­ordination of the Ca1 site. The residues involved are largely conserved with the equivalent sites in P19DFcnA and P5AFcnA. Inspection of the electron-density map based on the deposited structure-factor amplitudes of PDB entry 6m8n reveals that the crystal structure of P5AFcnA has a strong difference density peak located at an equivalent site to Ca1 in Mef1, which is likely to be an Na^+^ ion but could easily accommodate a Ca^2+^ ion, although this would need further investigation. The data suggest that there might be a level of conservation of this Ca^2+^-binding site in this subclass of fucoidanases, while in others, such as MfFcnA, FFA2, Fhf1 and Fhf2, no conservation seems to be present according to alignment. The importance of Ca^2+^ ions for Mef1 activity is likely to be related to the structural stability of the enzyme since the thermal stability increases when Ca^2+^ is present. The mechanistic role of Ca^2+^ coordination in GH107 fucoidanase enzymes has not previously been investigated and no importance of the Ca^2+^ ions has previously been suggested. Whether Ca^2+^ ions contribute similar stability in other GH107 fucoid­anase enzymes remains to be elucidated, but the prospect is supported by the results obtained for Mef2, where the *T*_m_ increased from 38 to 44°C on the addition of Ca^2+^ ions (Tran, Nguyen *et al.*, 2022 ▸).

The Mef1 crystals only formed when either CAPSO or CHES buffer molecules were present in the crystallization buffer, suggesting that these molecules influence the success of the crystallization process. Interestingly, both molecules were shown to have an inhibitory effect on the Mef1 fucoidanase and thus are likely to have stabilized locked states of the structures, which would favor crystallization. Clear peaks in the difference electron-density maps were found in the active site of Mef1, which we modeled as a CAPSO and an EG molecule. Although CAPSO is an inhibitor of the fucoidanase and is not a natural substrate, the sulfonate moiety of the CAPSO molecule was coordinated in the active site and its position could indicate the positions of the sulfate ions present in the natural fucoidan substrates, including the sulfated fucoidan from *F. evanescens*. However, molecular docking (Fig. 6 ▸) of a fucoidan hexasaccharide molecule model encompassing the tetrameric fucoidan product (Fig. 5 ▸ *c*) into the active site of Mef1 did not show complete congruence between the subsite positioning of the fucoidan sulfate groups and the positioning of the CAPSO sulfonate derived from the CAPSO crystallization data (Fig. 6 ▸). Still, the positioning of the sulfate groups of the fucoidan substrate is likely to contribute to the substrate selectivity of the Mef1 enzyme–substrate complex. The structural analysis of Mef1 thus hinted at a possible role of the positive charge of Arg35 in correct docking of the sulfated fucoidan substrate in the active-site groove and thus in the substrate selectivity of Mef1. However, sequence alignments with other GH107 fucoidanases (Supplementary Fig. S2) and structural alignment with P5AFcnA (PDB entry 6m8n) revealed Arg53 of P5AFcnA two residues away from Arg35 of Mef1, but the alignment could not establish the general significance of this arginine in P5AFcnA or in the other analyzed fucoidanases. Yet, the possibility that Arg35 is central for substrate recognition and substrate docking of Mef1 finds support in the early seminal work on the structure of *Alteromonas fortis* ι-carrageenase (PDB entry 1h80; now called *A. macleodii* 1,3-α-1,4-β-d-galactose-4-sulfate-3,6-anhydro-d-galactose-2-sulfate 4-galacto­hydrolase; Michel *et al.*, 2001 ▸). This work identified the possibility of salt-bridge formation to ester sulfates in the carrageenan substrate by two central arginine residues (Arg243 and Arg303) in the active site of the enzyme, thus proposing a conceivable function of the arginine residues in precisely placing the scissile bond in the sulfated carrageenan backbone for cleavage in the active site of the ι-carrageenase. Further work is required to establish whether Arg35 of Mef1 indeed has a similar function to help direct the cleavage and cleavage selectivity of sulfated fucoidan.

The water wire identified to span from the exterior of the Mef1 enzyme to the active site, closely passing the Ca1 site, might play an important role in the catalytic cycle of Mef1. During the hydrolysis of the carbohydrate backbone the excess proton needs to be allocated out of the active site, which for Mef1 could be through this newly identified water wire. Water wires for proton transport have been identified and experimentally verified in several biological systems, including, for example, photosystem II (Umena *et al.*, 2011 ▸) and the enzyme isatin hydrolase (Bjerregaard-Andersen *et al.*, 2014 ▸). Furthermore, in Mef1 the Ca1 site might be important for this water wire to be functional or it might be essential for proper stabilization of the structure of the water wire during catalysis, although this hypothesis needs further investigation.

The substrate specificities of the GH107 fucoidanase family vary, which is likely to reflect the broad range of chemical bonds, sulfate patterns and branched structures in fucoidan molecules from different species of brown seaweed. Fucoidans are present in seaweed cell walls, and their natural function is mainly considered to be to support the structural integrity of the cell walls and to provide a defense barrier system. Clearly, the complexity of fucoidan makes the polysaccharide quite resistant to enzymatic degradation, meaning that it requires an array of different enzymes for full degradation (Sichert *et al.*, 2020 ▸). It has been suggested that the robustness of fucoidan to microbial and enzymatic deconstruction, combined with its eventual slow degradation (as also shown by the extended reaction times for Mef1 employed in the present work), may imply that fucoidans, together with other complex seaweed glycans, may play a role in carbon sequestration and thus in carbon storage in the ocean, even after the death of the macroalgae (Bligh *et al.*, 2022 ▸). Indeed, brown macroalgae polysaccharide biomass is considered to be a likely CO_2_ carbon sink. Recent data on fucoidan exudate of *F. vesiculosus*, which appears to form an external mucilage barrier layer on this brown seaweed species, strongly suggest that this secreted fucoidan sequesters a significant amount of carbon, and thus that such extracellular fucoidan may contribute significantly to removal of CO_2_ from the atmosphere (Buck-Wiese *et al.*, 2023 ▸).

The bacterial strain called ‘*Lentimonas*’ sp. CC4, which was recently found to encode a very large number of diverse fucoidan-degrading enzymes, including several fucoidanases of family GH107 and various fucosidases of different GH families, as well as many sulfatases, has distinct genetic loci for the degradation of fucoidans from different types of brown seaweed (as well as for carrageenan from red seaweed), all collected on a megaplasmid (Sichert *et al.*, 2020 ▸). ‘*Lentimonas*’ sp. CC4 belongs to the phylum Verrucomicrobiota, whereas the marine bacterium *M. ecklonia*, originally described as *Flagellimonas eckloniae* (Bae *et al.*, 2007 ▸; García-López *et al.*, 2019 ▸), from which Mef1 originates, belongs to the Flavobactericeae family, a member of the large phylum Bacterioidota. The more elaborately studied *Bacteroides* species, which are commensals of the human gut, also belong to the Bacterioidota phylum. *Bacteroides* species are known to have (co-regulated) clusters of genes, organized in polysaccharide-utilization loci (PULs), that encode sets of enzymes for the degradation of complex polysaccharides, including charged polysaccharides such as pectins (Luis *et al.*, 2018 ▸). A PUL-like cluster of genes for fucoidan degradation, including genes encoding different α-l-fucosidases, has also recently been identified in the fucoidan-degrading marine bacterium *W. fucanolytica* CZ1127, which is also a member of the Bacteriodita phylum (Silchenko *et al.*, 2022 ▸). The discovery of Mef2, a new endo-α(1,3)-fucoidanase in GH107 (Tran, Nguyen *et al.*, 2022 ▸), and now the GH107 endo-α(1,4)-fucoidanase Mef1, both derived from *M. ecklonia*, invite further exploration of whether this organism may harbor more fucoidan-degradation enzymes in a fucoidan-degrading locus or more than one polysaccharide-utilization locus for fucoidan degradation, and of how the fucoidan-metabolizing machinery of this organism functions.

Although the selectivity of endo-fucoidanases is mainly tied to the type of backbone linkage that is being cleaved, *i.e.* α-1,4 or α-1,3, recent findings indicate that fucoidanase selectivity also involves specific branching and sulfation patterns in fucoidans (Silchenko *et al.*, 2017 ▸; Vuillemin *et al.*, 2020 ▸; Trang *et al.*, 2022 ▸; Tran, Nguyen *et al.*, 2022 ▸). NMR spectroscopy and real-time NMR data thus reveal that the Mef1 product formed has a distinct sulfation and acetylation pattern, namely sulfation at position C2 of each fucose unit and acetylation at the −2 site adjacent to the reducing end, while no LMP products containing 2,4-disulfations were observed (Fig. 5 ▸).

The apparent fucoidan acetylation pattern specificity of Mef1 corroborates the significance of backbone acetylation for the action of fucoidanases, as previously observed for Mef2 (Tran, Nguyen *et al.*, 2022 ▸), Fhf1 (Vuillemin *et al.*, 2020 ▸) and Fhf2 (Trang *et al.*, 2022 ▸). Notably, previous data show that Fhf1, Mef2 and Fhf2, like Mef1, tolerate acetylated residues near the cleavage site and thus catalyze the liberation of acetylated fragments from *F. evanescens* fucoidan (Trang *et al.*, 2022 ▸; Tran, Nguyen *et al.*, 2022 ▸). This is the first time that real-time NMR has been employed to monitor the hydrolysis of fucoidans by a fucoidanase, and we believe that this method is highly applicable to future fucoidanase studies. We conclude that the fucoidanase Mef1 catalyzes the cleavage of α(1,4)-glycosidic bonds within the structural motif [-3)-α-l-Fuc*p*2S-(1,4)-α-l-Fuc*p*2S-(1-]_*n*_, while leaving the structural motif [-3)-α-l-Fuc-2S-(1,4)-α-l-Fuc-2S-3Ac-(1-] intact.

Real-time reaction information was furthermore provided by FTIR observations combined with PARAFAC analysis. Such real-time methodologies hold promise for assisting in the characterization of fucoidanase action and aid in the development of suitable, well defined fucoidan products with specific, desirable bioactivities. The present study shows that Mef1 is a new promising fucoidanase enzyme for the production of specific fucoidan products suitable for future bioactivity studies.

## Supplementary Material

PDB reference: *Muricauda eckloniae* endo-α(1,4)-fucoidanase, 8bpd

Supplementary Tables and Figures. DOI: 10.1107/S2059798323008732/jc5061sup1.pdf

## Figures and Tables

**Table 1:** Data-collection and refinement statistics for Mef1 Values in parentheses are for the highest resolution shell.

Wavelength (Å)	0.9786
Resolution range (Å)	43.72–1.80 (1.864–1.800)
Space group	*P*6_3_
*a*, *b*, *c* (Å)	105.416, 105.416, 78.2472
α, β, γ (°)	90, 90, 120
Total reflections	313232 (30599)
Unique reflections	45842 (4540)
Multiplicity	6.8 (6.7)
Completeness (%)	99.87 (99.60)
Mean *I*/σ(*I*)	6.80 (0.48)
Wilson *B* factor (Å^2^)	25.27
*R*_merge_	0.1267 (1.01)
*R*_meas_	0.1369 (>1)
*R*_p.i.m._	0.05163 (0.4176)
CC_1/2_	0.996 (0.833)
CC*	0.999 (0.953)
Reflections used in refinement	45795 (4528)
Reflections used for *R*_free_	2259 (228)
*R*_work_	0.1639 (0.3618)
*R*_free_	0.1985 (0.3775)
CC(work)	0.973 (0.893)
CC(free)	0.965 (0.885)
No. of non-H atoms	
Total	3402
Macromolecules	2989
Ligands	121
Solvent	358
Protein residues	370
R.m.s.d., bond lengths (Å)	0.011
R.m.s.d., angles (°)	1.04
Ramachandran favored (%)	97.28
Ramachandran allowed (%)	2.72
Ramachandran outliers (%)	0.00
Rotamer outliers (%)	0.63
Average *B* factor (Å^2^)	
Overall	29.20
Macromolecules	27.89
Ligands	49.58
Solvent	37.00
No. of TLS groups	3

**Table 2:** Alignment of the catalytic amino acids in Mef1 and other GH107 members Red, the active-site aspartate (D); blue, the active-site histidine (H). Accession numbers are listed in Supplementary Fig. S2.

	Conserved motifs around the catalytic amino acids			
	I		II	
Mef1	175	ERFKGLADGYWL **D** HV	264	VDFTSG **H** PTP
P5AFcnA	189	ERFDGLVDGYWL **D** NS	270	MDFTNG **H** VTP
MfFcnA	214	QRYGDLIDAWCF **D** SA	288	DDYTFG **H** PFG
FFA2	225	MRYGDLIDAWCF **D** AA	302	EDYKFG **H** PFG
Fhf1	213	ERYGDLIDAWCF **D** SA	287	DDYTFG **H** PFG
Fhf2	215	MRYGDLIDAWCF **D** AA	291	EDYKFG **H** PFG
P19DFcnA	186	KRFKGLVDGFWL **D** NS	270	MDFTNG **H** VTP
Mef2	170	EVLKDYADGYWL **D** TV	254	QDFTNG **H** VTS
Fda1	214	LRYGSTIDGWWF **D** HS	267	DDYTFG **H** PTP
Fda2	283	LRYGTLIDGWWF **D** HS	336	EDFTGG **H** PTP
		-------*.::-*--		-*:.-**---

**Table 3:** Alignment of the −1 subsite in Mef1 and other GH107 members Blue, conserved amino acids previously suggested to be in the −1 subsite; orange, lack of conservation in several fucoidanases in one of the suggested −1 subsites; green, a conserved proline near the −1 subsite. Accession numbers are listed in Supplementary Fig. S2.

	Conserved motifs around the amino acid of the −1 subsite						
		1		2		3	Specificity
Mef1	122	GKKVIL **Y** I **A** TDGP	205	EVDPSVMIAS **N**	311	WM **P** MR--MK **W** TSP	α-(1,4)
P5AFcnA	137	GKKVIL **Y** L **N** SAGP	219	SVDPELTIAV **N**	318	WF **P** IR--NS **W** SGS	n.d.†
MfFcnA	141	GLRTEI **Y** V **N** SYNL	260	AGNPNAAIAF **N**	342	FF **P** KQSTTS **W** NAG	α-(1,4)
FFA2	148	GLKTEV **Y** V **N** SANL	270	AGNPDAAITF **N**	356	FF **P** KQSTTS **W** NDG	α-(1,4)
Fhf1	138	GLKTEI **Y** V **N** SYNL	258	AGNPNAAISF **N**	357	FF **P** KQSATS **W** NAG	α-(1,4)
Fhf2	138	GLKTEV **Y** V **N** SANL	260	AGNPNAAITF **N**	346	FF **P** KQSTTS **W** NDG	α-(1,4)
P19DFcnA	134	GKKVLL **Y** L **N** TAGP	219	DIDPSFAIGV **N**	318	WF **P** IR--FS **W** SGS	n.d.†
Mef2	121	DKKIIL **Y** I **S** TQYF	197	EVDPTAVVTT **N**	300	WF **P** VR--YR **W** HTS	α-(1,3)
Fda1	129	GIRVVA **Y** I **A** TQGP	241	AGNNDAAVAF **N**	314	FM **P** LQ--ES **W** NGG	α-(1,3)
Fda2	182	GIKVVA **Y** I **A** TQGP	310	AGNSNAAVSL **N**	383	FL **P** LQ--ET **W** NGG	α-(1,3)
		--:---*:-:---		--:----:--*		::*-:----*---	

† n.d., not determined.

**Table 4:** Alignment of the Ca^2+^ site in Mef1 and other GH107 members Green, amino acids predicted to be involved in Ca^2+^ coordination in Mef1. Accession numbers are listed in Supplementary Fig. S2.

	Mef1 calcium-site motifs			
	Ca2		Ca1	
Mef1	135	SARS--------GTRNN	239	VDSDGTNDPDG----RKYK
P5AFcnA	150	SMA---------EERGD	245	VDSDGLDDEDE----SDYK
MfFcnA	154	--LARIP-EDTQADYPD	271	---NSVGDREG-NP-----
FFA2	161	--LEWEAFGTPISEFPD	281	---NGIGDRDS-DP-----
Fhf1	149	--LARVP-DGIPAGYPD	267	---NSVGDREL-NP-----
Fhf2	151	--LQWEAFNGAPSEFPD	271	---NGIGDRDS-DP-----
P19DFcnA	147	THA---------ADRNS	245	VASDSIDDNDD----REYK
Mef2	134	A--R--------ADEDN	223	VASNGVSITGETSPKVGYN
Fda1	142	GMLKHGAEN--SMDEDD	253	---EGDK------------
Fda2	205	AMLKHGAER--SMDFDD	322	---LEGD------------

## References

[bb1] Adams, P. D., Afonine, P. V., Bunkóczi, G., Chen, V. B., Davis, I. W., Echols, N., Headd, J. J., Hung, L.-W., Kapral, G. J., Grosse-Kunstleve, R. W., McCoy, A. J., Moriarty, N. W., Oeffner, R., Read, R. J., Richardson, D. C., Richardson, J. S., Terwilliger, T. C. & Zwart, P. H. (2010). *Acta Cryst.* D**66**, 213–221. 10.1107/S0907444909052925 PMC2815670 20124702

[bb2] Ale, M. T. & Meyer, A. S. (2013). *RSC Adv.* **3**, 8131–8141.

[bb3] Bae, S. S., Kwon, K. K., Yang, S. H., Lee, H.-S., Kim, S.-J. & Lee, J.-H. (2007). *Int. J. Syst. Evol. Microbiol.* **57**, 1050–1054. 10.1099/ijs.0.64565-0 17473257

[bb4] Bilan, M. I., Grachev, A. A., Shashkov, A. S., Kelly, M., Sanderson, C. J., Nifantiev, N. E. & Usov, A. I. (2010). *Carbohydr. Res.* **345**, 2038–2047. 10.1016/j.carres.2010.07.009 20701899

[bb5] Bilan, M. I., Grachev, A. A., Ustuzhanina, N. E., Shashkov, A. S., Nifantiev, N. E. & Usov, A. I. (2002). *Carbohydr. Res.* **337**, 719–730. 10.1016/s0008-6215(02)00053-8 11950468

[bb6] Bilan, M. I., Grachev, A. A., Ustuzhanina, N. E., Shashkov, A. S., Nifantiev, N. E. & Usov, A. I. (2004). *Carbohydr. Res.* **339**, 511–517. 10.1016/j.carres.2003.10.028 15013388

[bb7] Bjerregaard-Andersen, K., Sommer, T., Jensen, J. K., Jochimsen, B., Etzerodt, M. & Morth, J. P. (2014). *J. Biol. Chem.* **289**, 21351–21359. 10.1074/jbc.M114.568824 PMC4118100 24917679

[bb8] Bligh, M., Nguyen, N., Buck-Wiese, H., Vidal-Melgosa, S. & Hehemann, J.-H. (2022). *Curr. Opin. Chem. Biol.* **71**, 102204. 10.1016/j.cbpa.2022.102204 36155346

[bb9] Bradford, M. M. (1976). *Anal. Biochem.* **72**, 248–254. 10.1016/0003-2697(76)90527-3 942051

[bb10] Buck-Wiese, H., Andskog, M. A., Nguyen, N. P., Bligh, M., Asmala, E., Vidal-Melgosa, S., Liebeke, M., Gustafsson, C. & Hehemann, J.-H. (2023). *Proc. Natl Acad. Sci. USA*, **120**, e2210561119. 10.1073/pnas.2210561119 PMC9910443 36584294

[bb60] Cao, H. T. T., Mikkelsen, M. D., Lezyk, M. J., Bui, L. M., Tran, V. T. T., Silchenko, A. S., Kusaykin, M. I., Pham, T. D., Truong, B. H., Holck, J. & Meyer, A. S. (2018). *Mar. Drugs.* **16**, 422. 10.3390/md16110422 PMC6267234 30388774

[bb11] Colin, S., Deniaud, E., Jam, M., Descamps, V., Chevolot, Y., Kervarec, N., Yvin, J. C., Barbeyron, T., Michel, G. & Kloareg, B. (2006). *Glycobiology*, **16**, 1021–1032. 10.1093/glycob/cwl029 16880504

[bb12] Davies, G. J., Wilson, K. S. & Henrissat, B. (1997). *Biochem. J.* **321**, 557–559. 10.1042/bj3210557 PMC1218105 9020895

[bb13] Drula, E., Garron, M. L., Dogan, S., Lombard, V., Henrissat, B. & Terrapon, N. (2022). *Nucleic Acids Res.* **50**, D571–D577. 10.1093/nar/gkab1045 PMC8728194 34850161

[bb14] Emsley, P., Lohkamp, B., Scott, W. G. & Cowtan, K. (2010). *Acta Cryst.* D**66**, 486–501. 10.1107/S0907444910007493 PMC2852313 20383002

[bb15] García-López, M., Meier-Kolthoff, J. P., Tindall, B. J., Gronow, S., Woyke, T., Kyrpides, N. C., Hahnke, R. L. & Göker, M. (2019). *Front. Microbiol.* **10**, 2083. 10.3389/fmicb.2019.02083 PMC6767994 31608019

[bb16] Harshman, R. A. & Lundy, M. E. (1994). *Comput. Stat. Data Anal.* **18**, 39–72.

[bb17] Laemmli, U. K. (1970). *Nature*, **227**, 680–685. 10.1038/227680a0 5432063

[bb18] Lahrsen, E., Liewert, I. & Alban, S. (2018). *Carbohydr. Polym.* **192**, 208–216. 10.1016/j.carbpol.2018.03.056 29691015

[bb19] Liu, Y., Yang, X., Gan, J., Chen, S., Xiao, Z.-X. & Cao, Y. (2022). *Nucleic Acids Res.* **50**, W159–W164. 10.1093/nar/gkac394 PMC9252749 35609983

[bb20] Luis, A. S., Briggs, J., Zhang, X., Farnell, B., Ndeh, D., Labourel, A., Baslé, A., Cartmell, A., Terrapon, N., Stott, K., Lowe, E. C., McLean, R., Shearer, K., Schückel, J., Venditto, I., Ralet, M.-C., Henrissat, B., Martens, E. C., Mosimann, S. C., Abbott, D. W. & Gilbert, H. J. (2018). *Nat. Microbiol.* **3**, 210–219. 10.1038/s41564-017-0079-1 PMC5784806 29255254

[bb21] Manns, D., Deutschle, A. L., Saake, B. & Meyer, A. S. (2014). *RSC Adv.* **4**, 25736–25746.

[bb22] McCoy, A. J., Grosse-Kunstleve, R. W., Adams, P. D., Winn, M. D., Storoni, L. C. & Read, R. J. (2007). *J. Appl. Cryst.* **40**, 658–674. 10.1107/S0021889807021206 PMC2483472 19461840

[bb23] Michel, G., Chantalat, L., Fanchon, E., Henrissat, B., Kloareg, B. & Dideberg, O. (2001). *J. Biol. Chem.* **276**, 40202–40209. 10.1074/jbc.M100670200 11493601

[bb24] Nguyen, T. T., Mikkelsen, M. D., Tran, V. H. N., Trang, V. T. D., Rhein-Knudsen, N., Holck, J., Rasin, A. B., Cao, H. T. T., Van, T. T. T. & Meyer, A. S. (2020). *Mar. Drugs*, **18**, 296. 10.3390/md18060296 PMC7344474 32498331

[bb25] Nielsen, M. S., Mikkelsen, M. D., Ptak, S. H., Hejbøl, E. K., Ohmes, J., Thi, T. N., Nguyen Ha, V. T., Fretté, X., Fuchs, S., Meyer, A., Schrøder, H. D. & Ding, M. (2022). *J. Biomed. Mater. Res.* **110**, 861–872. 10.1002/jbm.a.37334 34792851

[bb26] Ohmes, J., Mikkelsen, M. D., Nguyen, T. T., Tran, V. H. N., Meier, S., Nielsen, M. S., Ding, M., Seekamp, A., Meyer, A. S. & Fuchs, S. (2022). *Carbohydr. Polym.* **286**, 119286. 10.1016/j.carbpol.2022.119286 35337530

[bb27] Reardon, D. & Farber, G. K. (1995). *FASEB J.* **9**, 497–503. 10.1096/fasebj.9.7.7737457 7737457

[bb28] Robert, X. & Gouet, P. (2014). *Nucleic Acids Res.* **42**, W320–W324. 10.1093/nar/gku316 PMC4086106 24753421

[bb29] Sichert, A., Corzett, C. H., Schechter, M. S., Unfried, F., Markert, S., Becher, D., Fernandez-Guerra, A., Liebeke, M., Schweder, T., Polz, M. F. & Hehemann, J.-H. (2020). *Nat. Microbiol.* **5**, 1026–1039. 10.1038/s41564-020-0720-2 32451471

[bb30] Silchenko, A. S., Rubtsov, N. K., Zueva, A. O., Kusaykin, M. I., Rasin, A. B. & Ermakova, S. P. (2022). *Arch. Biochem. Biophys.* **728**, 109373. 10.1016/j.abb.2022.109373 35940339

[bb31] Silchenko, A. S., Ustyuzhanina, N. E., Kusaykin, M. I., Krylov, V. B., Shashkov, A. S., Dmitrenok, A. S., Usoltseva, R. V., Zueva, A. O., Nifantiev, N. E. & Zvyagintseva, T. N. (2017). *Glycobiology*, **27**, 254–263. 10.1093/glycob/cww138 28031251

[bb32] Takayama, M., Koyama, N., Sakai, T. & Kato, I. (2007). US Patent US6489155B1.

[bb33] Teufel, F., Almagro Armenteros, J. J., Johansen, A. R., Gíslason, M. H., Pihl, S. I., Tsirigos, K. D., Winther, O., Brunak, S., von Heijne, G. & Nielsen, H. (2022). *Nat. Biotechnol.* **40**, 1023–1025. 10.1038/s41587-021-01156-3 PMC9287161 34980915

[bb34] Thinh, P. D., Menshova, R. V., Ermakova, S. P., Anastyuk, S. D., Ly, B. M. & Zvyagintseva, T. N. (2013). *Mar. Drugs.* **11**, 1456–1476. 10.3390/md11051456 PMC3707154 23648551

[bb35] Tran, V. H. N., Nguyen, T. T., Meier, S., Holck, J., Cao, H. T. T., Van, T. T. T., Meyer, A. S. & Mikkelsen, M. D. (2022). *Mar. Drugs*, **20**, 305. 10.3390/md20050305 PMC9147238 35621956

[bb36] Tran, V. H. N., Perna, V., Mikkelsen, M. D., Nguyen, T. T., Trang, V. T. D., Baum, A., Cao, H. T. T., Van, T. T. T. & Meyer, A. S. (2022). *Enzyme Microb. Technol.* **158**, 110035. 10.1016/j.enzmictec.2022.110035 35489196

[bb37] Trang, V. T. D., Mikkelsen, M. D., Vuillemin, M., Meier, S., Cao, H. T. T., Muschiol, J., Perna, V., Nguyen, T. T., Tran, V. H. N., Holck, J., Van, T. T. T., Khanh, H. H. N. & Meyer, A. S. (2022). *Front. Plant Sci.* **13**, 823668. 10.3389/fpls.2022.823668 PMC8847386 35185990

[bb61] Umena, Y., Kawakami, K., Shen, J. & Kamiya, N. (2011). *Nature*, **473**, 55–60. 10.1038/nature09913 21499260

[bb38] Vickers, C., Liu, F., Abe, K., Salama-Alber, O., Jenkins, M., Springate, C. M. K., Burke, J. E., Withers, S. G. & Boraston, A. B. (2018). *J. Biol. Chem.* **293**, 18296–18308. 10.1074/jbc.RA118.005134 PMC6254363 30282808

[bb39] Vuillemin, M., Silchenko, A. S., Cao, H. T. T., Kokoulin, M. S., Trang, V. T. D., Holck, J., Ermakova, S. P., Meyer, A. S. & Mikkelsen, M. D. (2020). *Mar. Drugs*, **18**, 562. 10.3390/md18110562 PMC7698502 33213084

[bb40] Wang, Y., Xing, M., Cao, Q., Ji, A., Liang, H. & Song, S. (2019). *Mar. Drugs*, **17**, 183. 10.3390/md17030183 PMC6471298 30897733

[bb41] Yang, X., Liu, Y., Gan, J., Xiao, Z.-X. & Cao, Y. (2022). *Brief. Bioinform.* **23**, bbac087. 10.1093/bib/bbac087 35289358

[bb42] Zayed, A., Avila-Peltroche, J., El-Aasr, M. & Ulber, R. (2022). *Mar. Drugs*, **20**, 412. 10.3390/md20070412 PMC9319086 35877705

[bb43] Zvyagintseva, T. N., Usoltseva, R. V., Shevchenko, N. M., Surits, V. V., Imbs, T. I., Malyarenko, O. S., Besednova, N. N., Ivanushko, L. A. & Ermakova, S. P. (2021). *Carbohydr. Polym.* **273**, 118551. 10.1016/j.carbpol.2021.118551 34560963

