# Supplementary material for: Structural and functional characterization of the novel endo-α(1,4)-fucoidanase Mef1 from the marine bacterium Muricauda eckloniae
Source: Acta Crystallogr D Struct Biol. 2023 Oct 25;79(Pt 11):1026–43. doi: 10.1107/S2059798323008732 (PMC10619423; doi:10.1107/S2059798323008732)
Supplement: Supplementary file 1 [file d-79-01026-sup1.pdf]

STRUCTURAL BIOLOGY

Volume 79 (2023)

Supporting information for article:

Structural and functional characterization of the novel endo-α(1,4)-fucoidanase Mef1 from the marine bacterium *Muricauda eckloniae*

Maria Dalgaard Mikkelsen, Vy Ha Nguyen Tran, Sebastian Meier, Thuan Thi Nguyen, Jesper Holck, Hang Thi Thuy Cao, Tran Thi Thanh Van, Pham Duc Thinh, Anne S. Meyer and Jens Preben Morth

Table S1 Monomer compositions of the fucoidans that Mef1 was found to be able to degrade

The data are given in %mol (relative level) of total carbohydrates analyzed in each fucoidan fraction, with total sulfate (SO_4^{2-}) calculated as %weight (wt%) of the total (data adapted from Tran, Nguyen *et al.*, 2022).

Monomer category		*S. polycystum*	*S. oligocystum*	*T. ornata*	*S. mcclurei*
Neutral monosaccharides (%mol)	Fucose	46.7 ± 4.5	66.4 ± 3.4	85.6 ± 0.3	78.5 ± 1.7
	Rhamnose	0.5 ± 0.4	0.8 ± 0.6	0.0 ± 0.0	0.3 ± 0.0
	Galactose	26.1 ± 4.9	24.3 ± 2.3	9.9 ± 0.5	15.4 ± 0.5
	Glucose	1.3 ± 0.0	0.7 ± 0.3	0.2 ± 0.0	0.1 ± 0.1
	Xylose	18.3 ± 2.6	3.8 ± 0.1	0.8 ± 0.0	2.8 ± 0.3
	Mannose	0.00 ± 0.0	1.1 ± 0.0	0.2 ± 0.1	0.0 ± 0.0
Uronic acids (%mol)	Guluronic acid	0.7 ± 0.2	0.1 ± 0.0	0.2 ± 0.0	0.1 ± 0.1
	Glucuronic acid	0.6 ± 0.1	1.0 ± 0.1	0.5 ± 0.2	0.5 ± 0.2
	Mannuronic acid	5.2 ± 0.5	1.8 ± 0.0	2.5 ± 0.0	2.0 ± 2.1
Sulfate ($SO_4^{2-)}$ (wt%)		24.6 ± 0.8	27.7 ± 1.8	(na)	31.4 ± 2.6

na = not analyzed

Table S2 Composition of fucoidan fractions F3 (FeF3) and F4 (FeF4) of *F. evanescens*

Data are given in %mol (relative level) of total carbohydrates analyzed in each fucoidan fraction, with total sulfate (SO_4^{2-}) calculated as %weight (wt%) of the total.

		FeF3	FeF4
Neutral monosaccharides (%mol)	Fucose	87.3 ± 0.7	90.4 ± 0.6
	Rhamnose	0.48 ± 0.11	0.27 ± 0.05
	Galactose	8.47 ± 0.33	7.32 ± 0.26
	Glucose	0.32 ± 0.04	0.52 ± 0.09
	Xylose	1.59 ± 0.38	0.78 ± 0.18
	Mannose	0.36 ± 0.08	0.14 ± 0.04
Uronic acids (%mol)	Glucuronic acid	0.68 ± 0.14	0.05 ± 0.04
	Guluronic acid	(nd)	(nd)
	Mannuronic acid	0.74 ± 0.16	0.24 ± 0.06
Sulfate ($SO_4^{2-)}$ (wt%)		51.5 ± 6.2	55.2 ± 5.9

nd = not detectable

Table S3 Yields and monosaccharide compositions of the Mef1 hydrolyzed products from *F. evanescens* (FeF4), *i.e.*, medium molecular weight fucoidan products (MMP) and low molecular weight fucoidans (LMP).

Yields are given as % weight (wt%) of the FeF4 fraction. The compositional data are given in %mol (relative level) of total carbohydrates analyzed in the fucoidan fractions, with total sulfate (SO_4^{2-}) calculated as %weight (wt%) of the total.

		MMP	LMP
	Yields (wt%)	61.8	38.2
Neutral monosaccharides (%mol)	Fucose	89.7 ± 0.8	97.9 ± 1.0
	Rhamnose	2.22 ± 0.59	0.22 ± 0.01
	Galactose	6.18 ± 0.24	0.89 ± 0.00
	Glucose	1.16 ± 0.14	0.06 ± 0.00
	Xylose	1.61 ± 0.11	0.93 ± 0.00
	Mannose	(nd)	(nd)
Uronic acids (%mol)	Glucuronic acid	0.98 ± 0.09	(nd)
	Mannuronic acid	(nd)	(nd)
	Guluronic acid	(nd)	(nd)
Sulfate (SO_4^{2-}) (wt%)		48.9 ± 2.98	30.3 ± 2.23

nd = not detectable

Table S4 Percent identity matrix of the catalytic D1 domain of selected GH107 family members.

*indicates amino acid sequence numbers used for the analysis

	Mef1	P5A FcnA	P19DF cnA	Mef2	Mf FcnA	FFA1	FWf4	FWf3	FFA2	Fhf2	Fhf1	Fp273	FWf1	Fp277	Fp279	FWf2	Fda1	Fda2	SVI_0379
**Mef1/24-394***	*100*	**51**	**49**	**30**	**19**	**20**	**21**	**19**	**21**	**21**	**21**	**18**	**18**	**19**	**21**	**17**	**17**	**17**	**20**
P5AFcnA/36-393*	**51**	*100*	70	27	22	20	20	19	22	21	21	19	21	21	22	18	18	17	22
P19DFcnA/31-393*	**49**	70	*100*	26	20	21	21	20	22	22	21	21	21	20	23	19	20	18	21
Mef2/22-383*	**30**	27	26	*100*	19	19	19	18	19	19	18	16	16	20	18	19	16	16	18
MfFcnA/34-418*	**19**	22	20	19	*100*	67	63	43	56	57	57	35	46	40	41	38	19	20	25
FFA1/31-415*	**20**	20	21	19	67	*100*	64	42	58	56	57	34	46	41	41	39	17	17	23
FWf4/37-421*	**21**	20	21	19	63	64	*100*	65	52	52	57	37	46	43	41	41	19	18	24
FWf3/213-589*	**19**	19	20	18	43	42	65	*100*	42	41	41	57	39	39	37	46	19	18	20
FFA2/41-433*	**21**	22	22	19	56	58	52	42	*100*	83	61	34	46	41	41	39	18	18	22
Fhf2/31-439*	**21**	21	22	19	57	56	52	41	83	*100*	62	34	44	41	41	40	19	18	22
Fhf1/29-447*	**21**	21	21	18	57	57	57	41	61	62	*100*	34	45	41	39	38	16	17	21
Fp273/234-617*	**18**	19	21	16	35	34	37	57	34	34	34	*100*	36	42	40	43	17	17	20
FWf1/15-439*	**18**	21	21	16	46	46	46	39	46	44	45	36	*100*	53	49	45	19	18	20
Fp277/26-435*	**19**	21	20	20	40	41	43	39	41	41	41	42	53	*100*	62	45	20	19	22
Fp279/40-449*	**21**	22	23	18	41	41	41	37	41	41	39	40	49	62	*100*	40	20	19	20
FWf2/243-679*	**17**	18	19	19	38	39	41	46	39	40	38	43	45	45	40	*100*	18	15	22
Fda1/23-372*	**17**	18	20	16	19	17	19	19	18	19	16	17	19	20	20	18	*100*	72	30
Fda2/84-439*	**17**	17	18	16	20	17	18	18	18	18	17	17	18	19	19	15	72	*100*	29
SVI_0379/26-367*	**20**	22	21	18	25	23	24	20	22	22	21	20	20	22	20	22	30	29	*100*

Table S5 Tm of Mef1 at different conditions

Conditions	Tm (°C)
Mef1 + Ca^{2+}	43.2 ± 0.03
Mef1 (EDTA)	40.0 ± 0.18
Mef1 (EDTA) + fucoidan	43.4 ± 0.11
Mef1 + Ca^{2+} + fucoidan	46.0 ± 0.01

Table S6 ^{1}H and ^{13}C NMR data for the Mef1 oligosaccharide (δ $^{1}H/^{13}C$, ppm)

Residue	H1/C1	H2/C2	H3/C3	H4/C4	H5/C5	H6/C6
A	5.33/95.3	4.65/73.9	4.37/68.6	3.90/73.4	4.60/68.5	1.24/16.6
B	5.25/100.1	4.58/74.8	4.22/73.8	4.13/70.2	4.41/68.4	1.29/16.8
C	5.36/95.2	4.69/74.4	5.39/71.0 (OAc)	4.13/80.6	4.58/69.0	1.38/16.9
D(α-anomer)	5.49/91.9	4.55/74.7	4.09/74.1	4.09/70.1	4.23/67.3	1.24/16.6

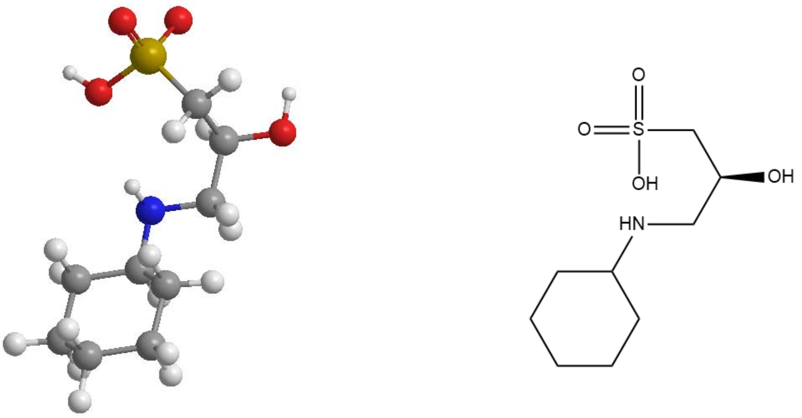

Figure S1 Molecular structural displays of CAPSO in 3D and 2D presentations, respectively. Systematic name: 3-(cyclohexyl-amino)-2-hydroxy-1-propane sulfonic acid; Molecular formula: $C_9H_{19}NO_4S$; SMILES: C1CCC(CC1)NCC(CS(=O)(=O)O)O.

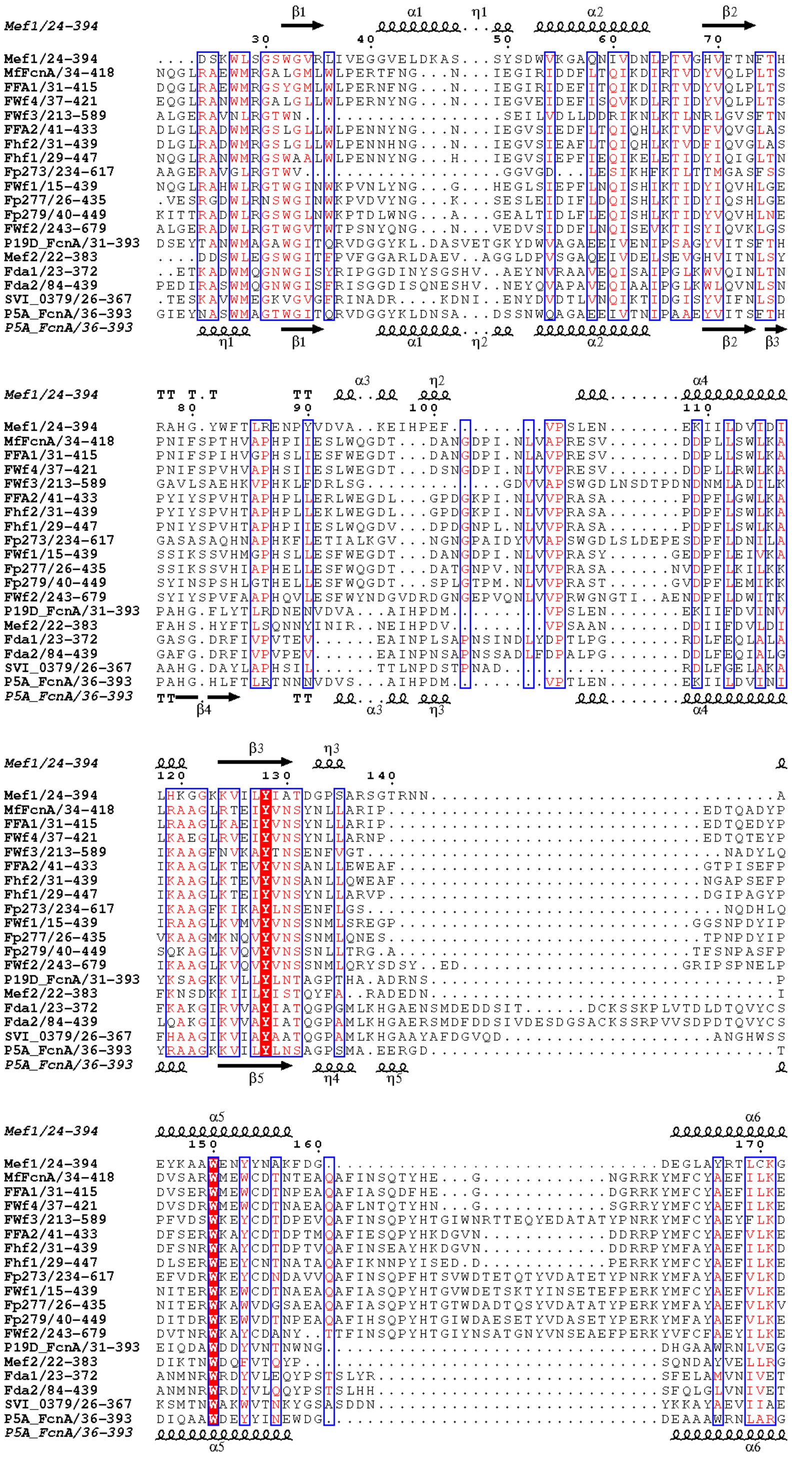

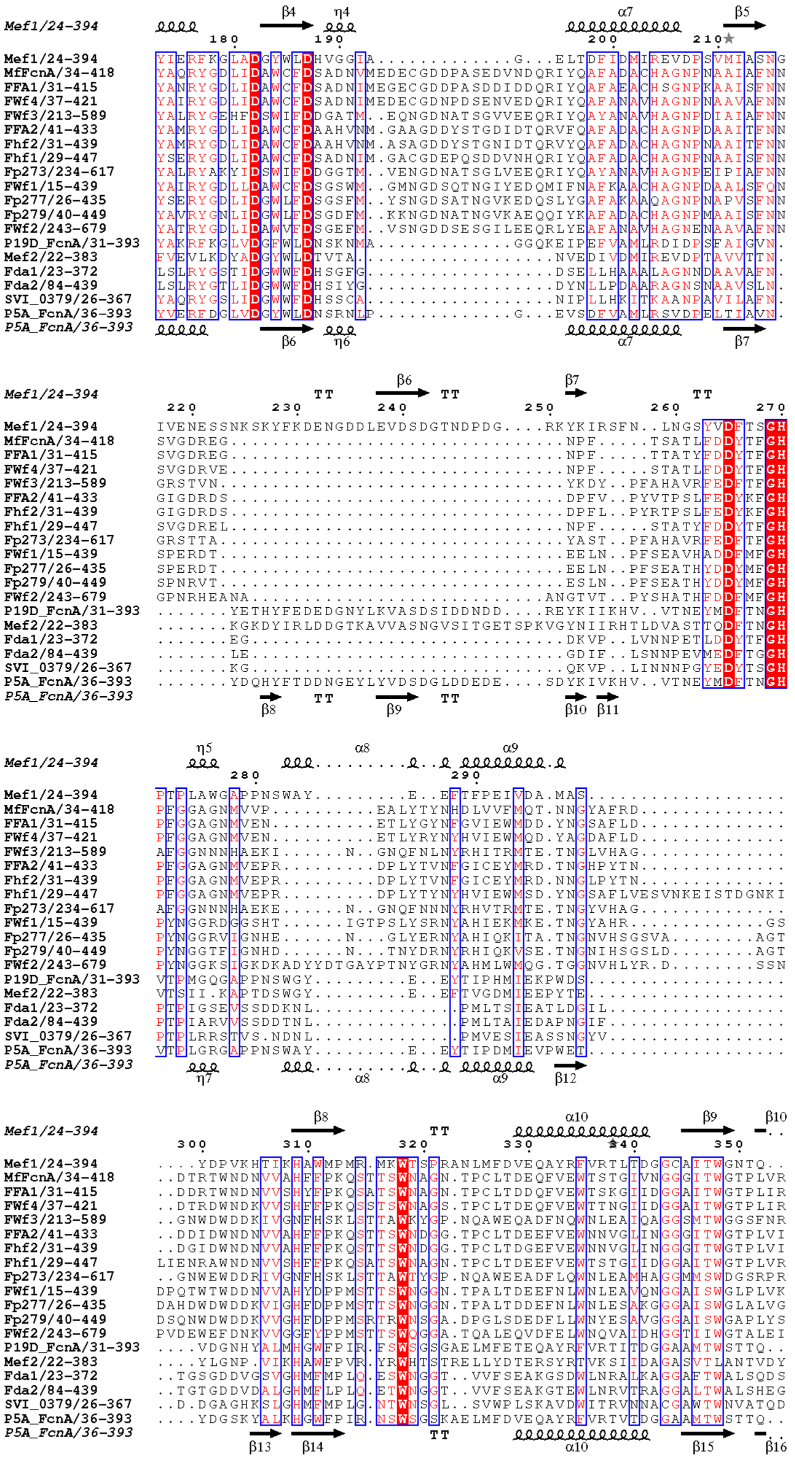
Mef1/24-394
MfFcnA/34-418
FFA1/31-415
FWf4/37-421
FWf3/213-589
FFA2/41-433
Fhf2/31-439
Fhf1/29-447
Fp273/234-617
FWf1/15-439
Fp277/26-435
Fp279/40-449
FWf2/243-679
P19D_FcnA/31-393
Mef2/22-383
Fda1/23-372
Fda2/84-439
SVI_0379/26-367
P5A_FcnA/36-393

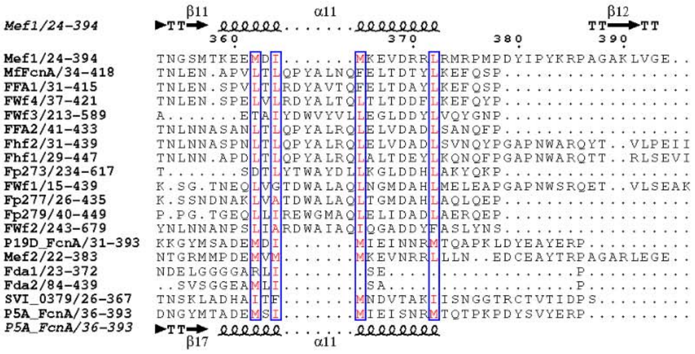

Figure S2 Multiple sequence alignment of the D1 domain of selected GH107 fucoidanases. The alignment is of the D1 catalytic domain using the sequence as indicated after each sequence name. The alignment includes the X-ray crystallographically determined α-helixes and β-sheets in Mef1 above and P5AFcnA below. Conserved amino acids are highlighted. Accession numbers: Mef1 (Genbank: AAY42_01290), P5AFcnA (GenBank: AYF59291.1), P19DFcnA (GenBank: AYF59292.1), FWf3 (Genbank: ANW96115.1), Fp273 (GenBank: AYC81238.1), FFA2 (RefSeq: WP_057784219.1), Fhf2 (Genbank: UQB70640.1 and UQB70641.1), Fhf1 (Genbank: UQB70638.1 and UQB70639.1), FWf4 (Genbank: ANW96116.1), MfFcnA (GenBank: CAI47003.1), FFA1 (RefSeq: WP_057784217.1), FWf2 (Genbank: ANW96098.1), Fp279 (GenBank: AYC81240.1), FWf1 (Genbank: ANW96097.1), Fp277 (GenBank: AYC81239.1), SVI_0379 (GenBank: BAJ00350.1), Fda1 (GenBank: AAO00508.1), Fda2 (GenBank: AAO00509.1), and Mef2 (Genbank: URS64324.1).

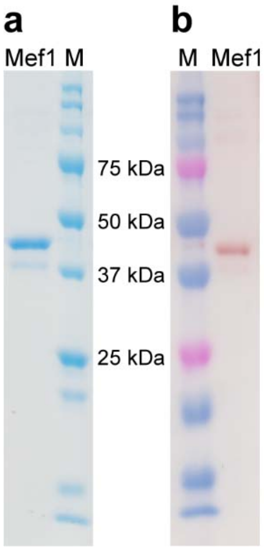

Figure S3 a) SDS-PAGE and b) western blot of the purified recombinant Mef1 fucoidanse. Lane 2 and 1 (indicated with M) are molecular weight marker proteins. The expected molecular weight of the purified enzyme was 45 kDa.

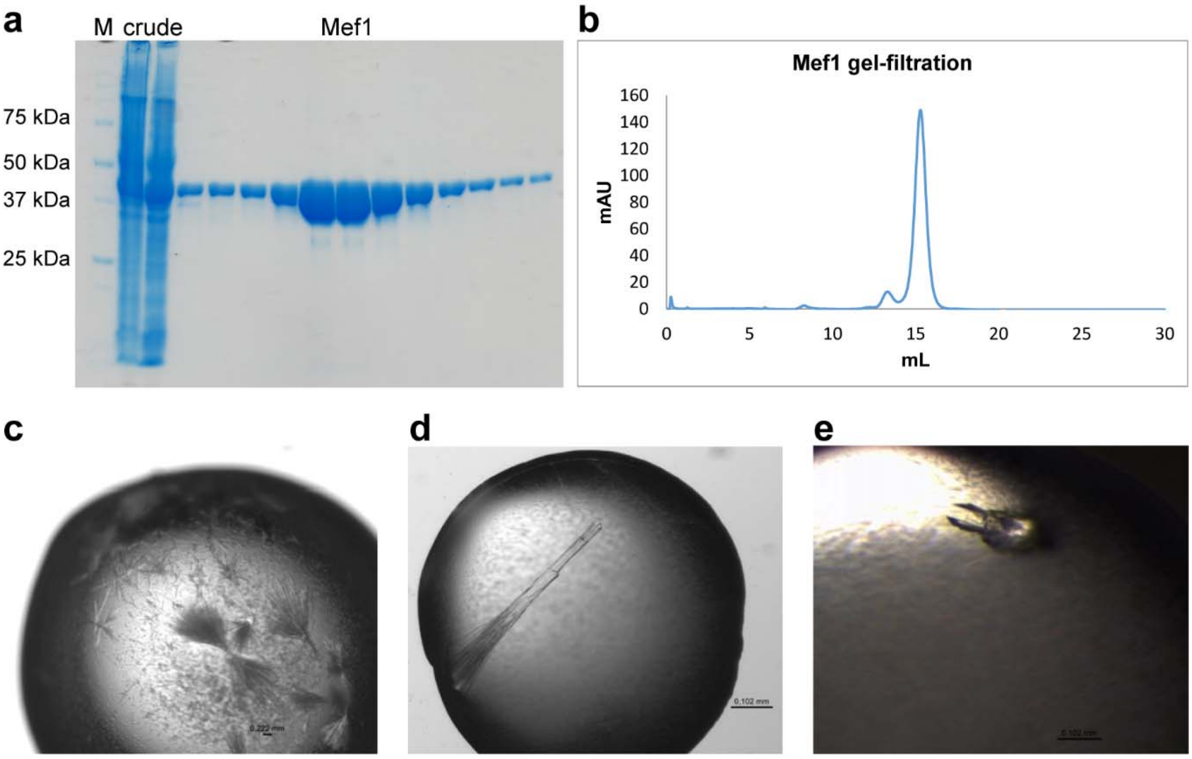

Figure S4 Concentration, SEC analysis and crystal formation of Mef1. a) SDS-PAGE of Mef1 in crude extracts and in elutions after gel filtration. M: protein marker. b) Analytical SEC gel-filtration of the Mef1 fucoidanase comparison with the standard shows that: the elution volume was 15.18 ml corresponding to 42 kDa; hence showing that Mef1 is a natural monomer. This result is consistent with the SDS-PAGE and gene prediction results. c-e) Displays of Mef1 crystals.

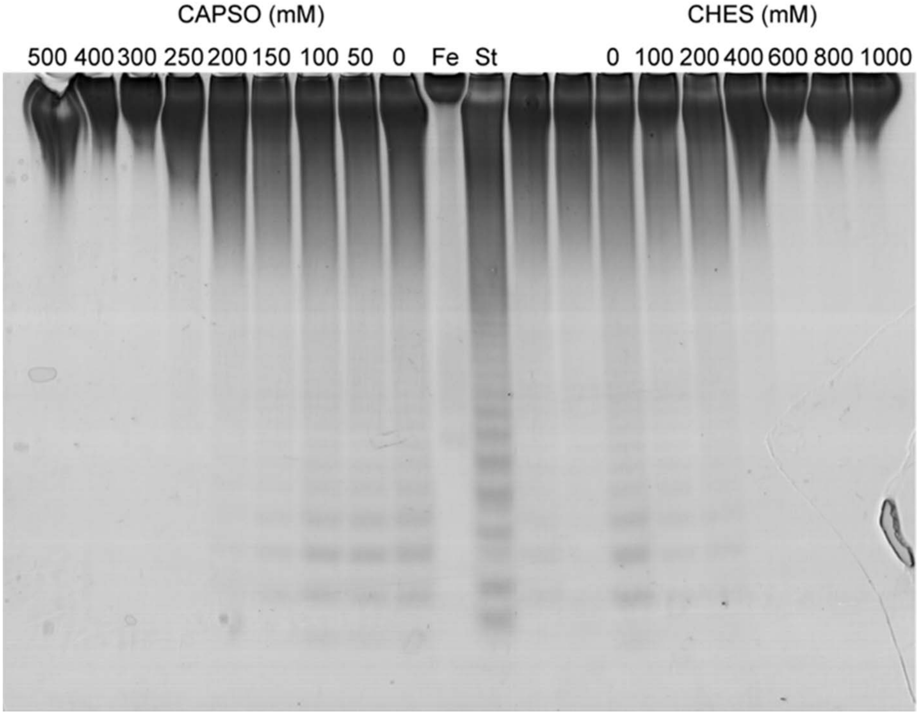

Figure S5 Inhibition of the Mef1 fucoidanase with CAPSO and CHES by C-PAGE. Mef1 was pre-incubated with different concentrations of CAPSO (mM as indicated) and CHES (mM as indicated) at 25 °C for 1 h. The enzyme was incubated with 0.9 % w/v fucoidan from *F. evanescens* (Fe) in presence of 10 mM Ca^{2+}, at pH 9, 37 °C for 2 h. St) An oligosaccharide hydrolysate standard obtained after enzymatic reaction of FFA2 on Fe fucoidan.

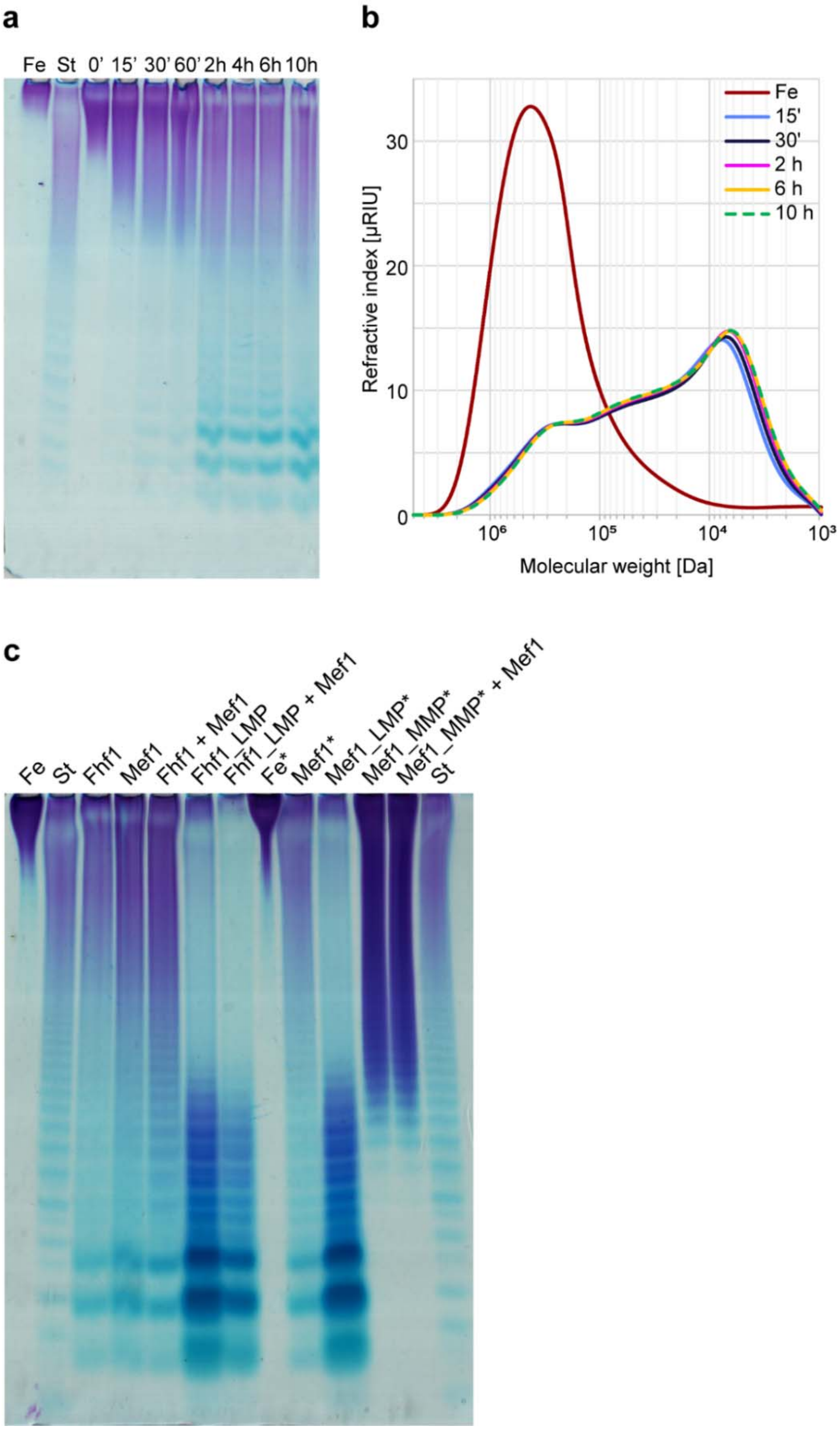

Figure S6 a) C-PAGE track of a time-course assay; time of reaction indicated as 0, 15' (min), 30' (min), 60' (min), 2 h, 4 h, 6 h, and 10 h; b) HP-SEC chromatogram of selected time-course experiments (0, 15', 30', 2h, 6h, and 10h). c) C-PAGE of Mef1 and Fhf1 activity on fucoidan from *F. evanescens* (*: fraction F4), including the EtOH separated low molecular weight products (LMP) and medium molecular products (MMP). St: An oligosaccharides standard of FFA2 acting on Fe fucoidan.

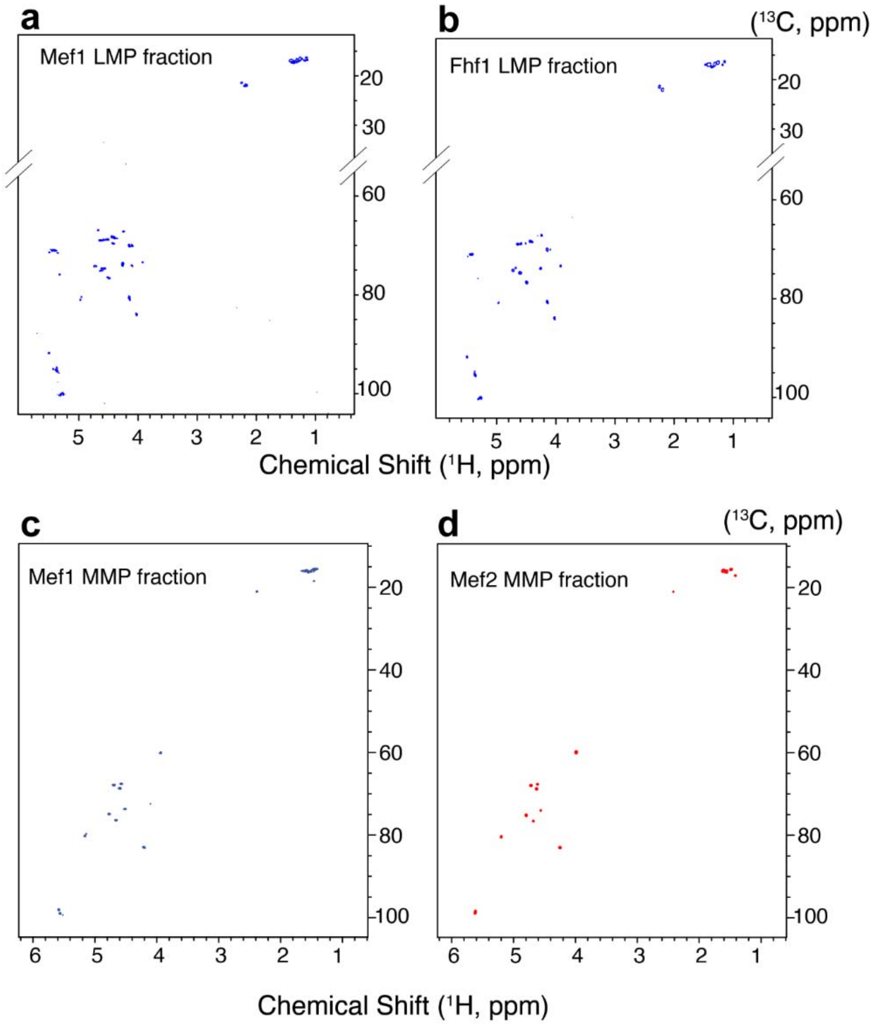

Figure S7 ^{1}H-^{13}C NMR spectroscopy was used as a fingerprint to show that identical fucoidan fragments predominate upon Mef1 and Fhf1 cleavage of fucoidan: Comparison of the LMP fractions upon *F. evanescens* fucoidan degradation with Mef1 (a) and Fhf1 (b). Comparison of the MMP fractions upon *F. evanescens* fucoidan degradation with Mef1 (c) and Mef2 (d) likewise indicates similar activity. Sulfated fucose residues in these fragments are partially acetylated (signals at $\delta^{1}H \approx 2.1$ ppm, $\delta^{13}C \approx 21.0$ ppm) in the LMP fraction (a and b), less so in the MMP fraction (c and d).

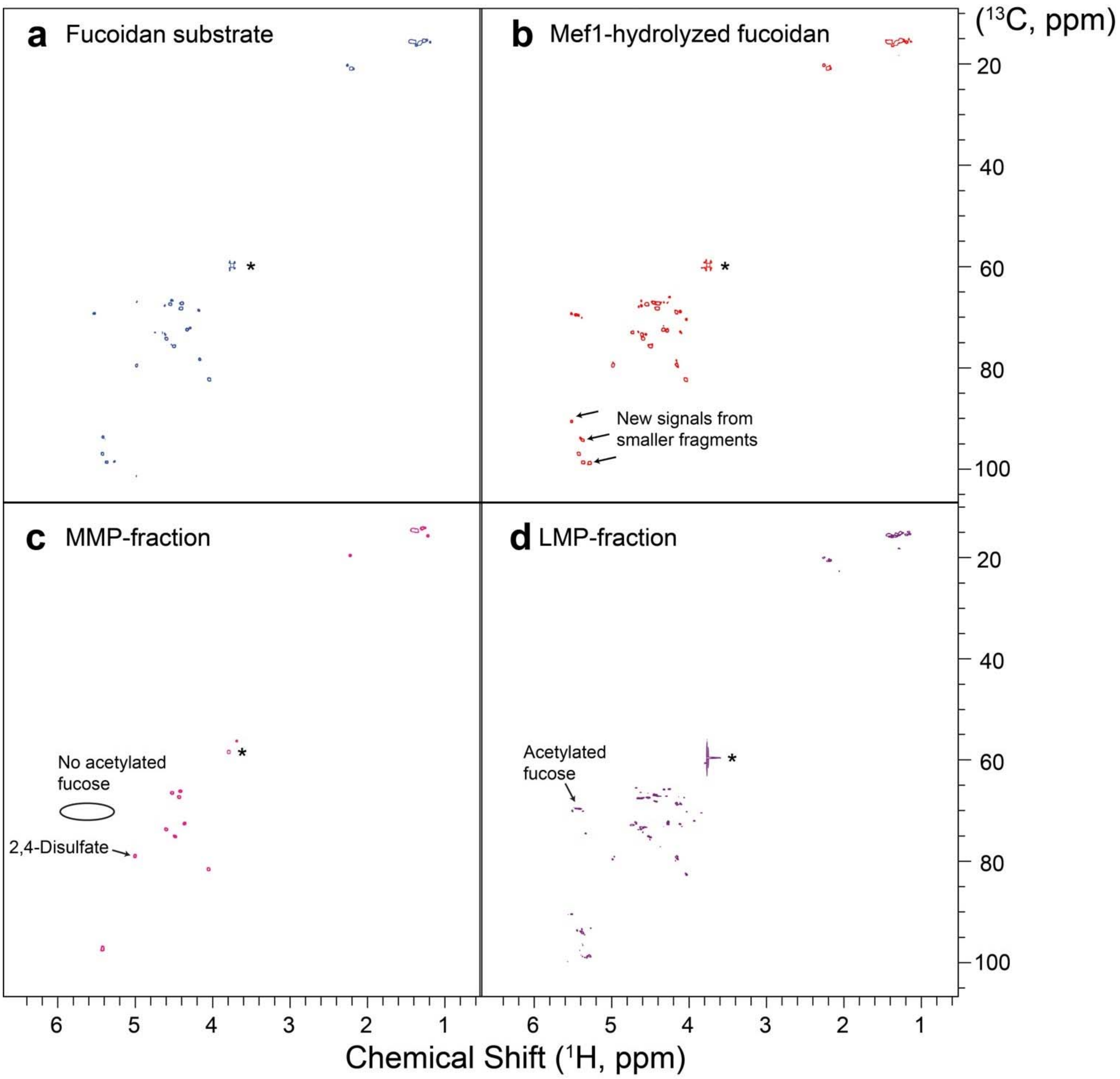

Figure S8 ^{1}H-^{13}C NMR spectra of the *F. evanescens* fucoidan hydrolysis and products by Mef1, specifically of (a) the F. evanescens fucoidan substrate, (b) the Mef1 degraded F. evanescens fucoidan, (c) The MMP and (d) the LMP hydrolysis products after Mef1 hydrolysis of *F. evanescens* fucoidan. Arrows in subpanel (b) point to the formation of reducing ends and fucosyl units in smaller fragments akin to the LMP fraction (new signals from smaller fragments). The arrows in subpanel (c) points to the presence of 2,4-disulfated units in the MMP fraction (which were not present in the LMP fraction in (d)), while the circle indicates the lack of acetylated fucoidan in the MMW fraction after Mef1 hydrolysis. In (d), an arrow indicates the presence of acetylated fucosyl units in the LMP fraction (which were not present in the HMP fraction in (c)). The asterisk indicates a signal of the Tris-HCl buffer (10 mM, pH 8).

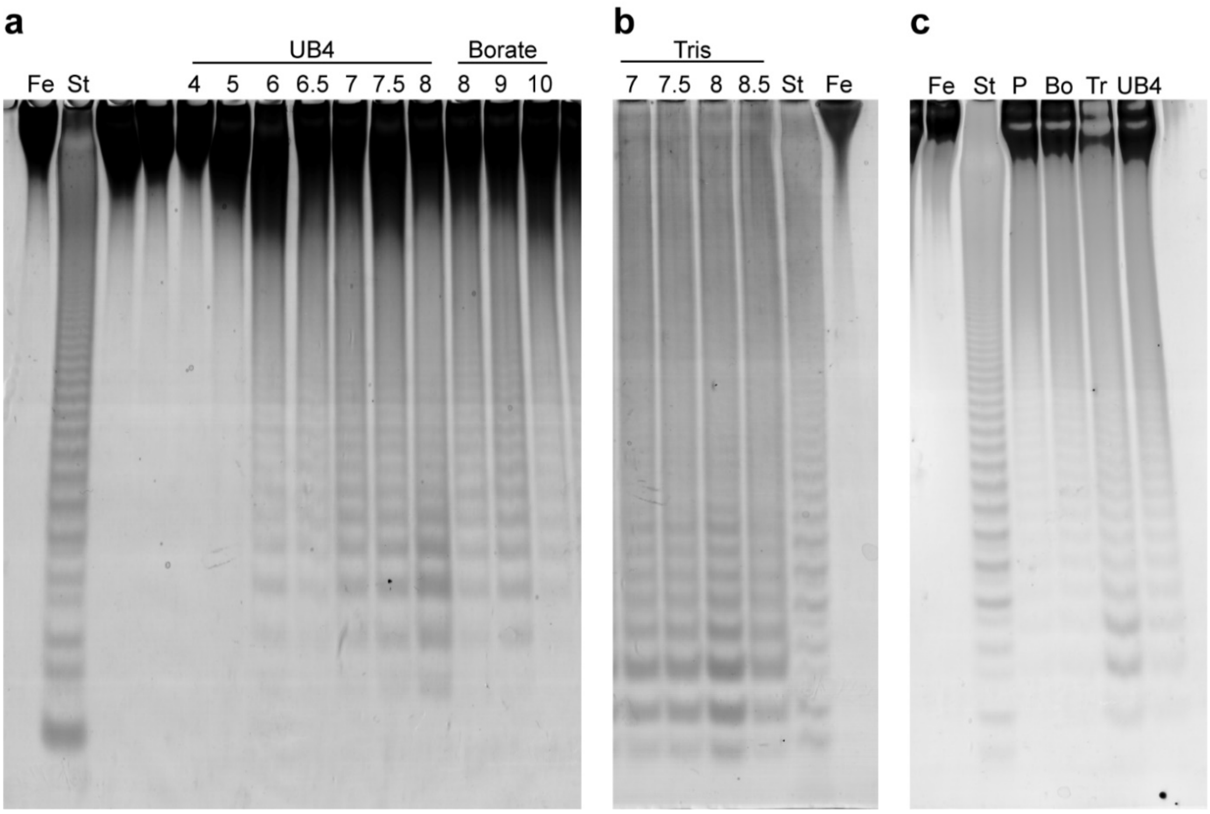

Figure S9 Assessment of pH optimum of Mef1 by C-PAGE. a) Effect of pH 4.0, 5.0, 6.0, 7.0, 7.4, 8.0, 9.0, and 10.0 on fucoidanase activity in overlapping buffers, UB4 and borate buffer respectively. b) pH optimum determination in Tris buffer at the different pH 7.0, 7.5, 8.0 and 8.5. c) The influence of different buffers, including sodium phosphate buffer (PP), borate buffer (Bo), Tris-HCl (Tr) and UB4 at pH 8.0. St) A hydrolysate standard obtained after enzymatic reaction of FFA2 on Fe fucoidan.

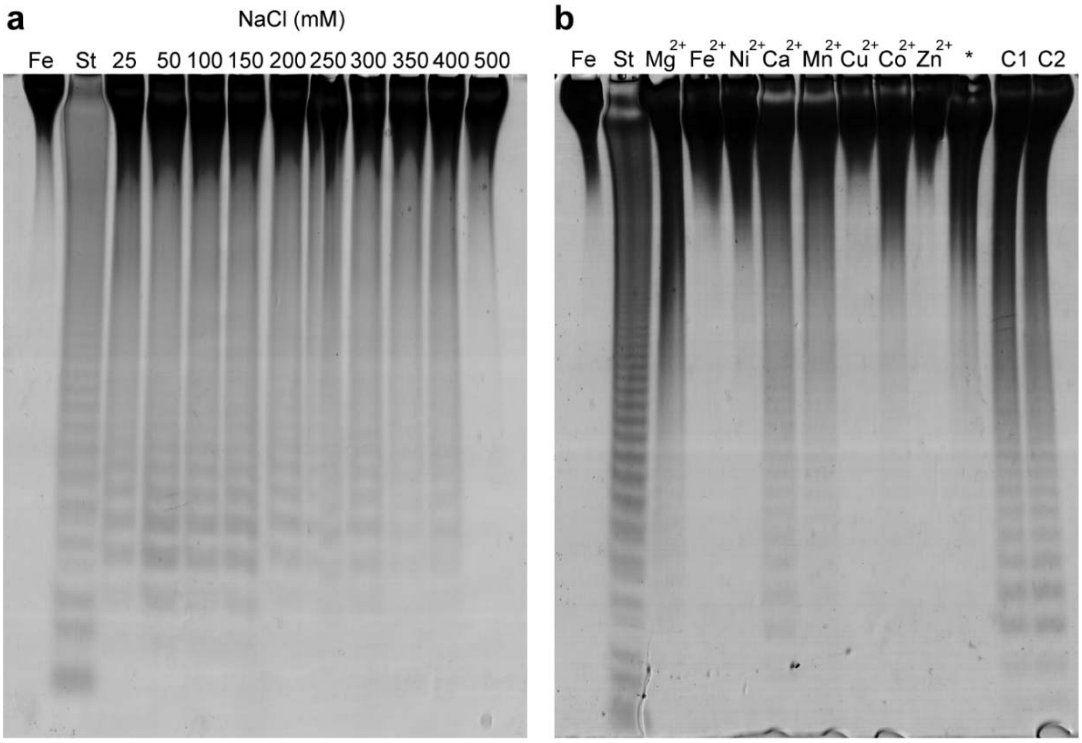

Figure S10 Influence of NaCl and different divalent cations on Mef1 activity assessed by C-PAGE. a) Mef1 activity using increasing concentrations of NaCl ranging from 25 to 500 mM. b) Effect of the divalent cations Ca^{2+}, Mg^{2+}, Mn^{2+}, Cu^{2+}, Fe^{2+}, Zn^{2+}, Co^{2+}, Ni^{2+} at 10 mM and (*) without cation after EDTA (2 mM) treatment on fucoidanase activity. A PD10 column was used to remove EDTA. The reaction was carried out at 10 mM Ca^{2+} for further experiments. C1: reaction before EDTA treatment, i.e., in presence of 10 mM Ca^{2+}, C2: reaction before EDTA treatment, (no Ca^{2+} added). St) A hydrolysate standard obtained after enzymatic reaction of FFA2 on Fe fucoidan.

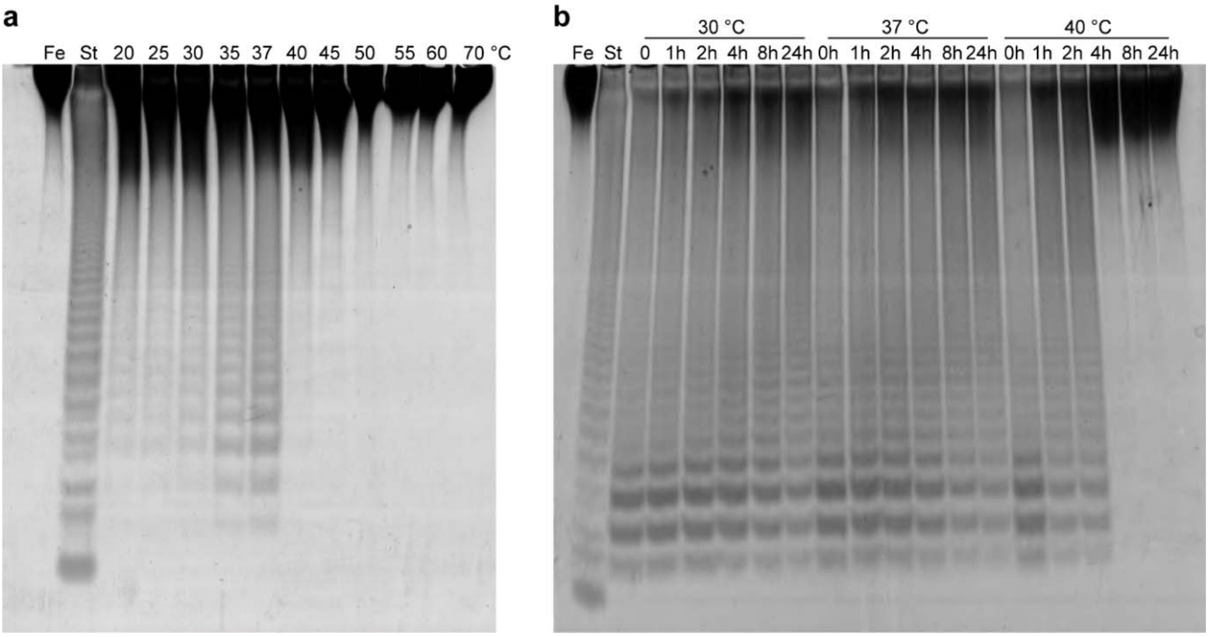

Figure S11 Temperature optimum and thermostability of Mef1 by C-PAGE. a) Influence of assay temperatures ranging from 20 - 70 °C on Mef1 activity on Fe fucoidan. b) Thermal stability of the Mef1 fucoidanase. Mef1 was pre-incubated without substrate at 30, 37 and 40 °C for 0-24 h as indicated. The enzyme assay was then performed by addition of substrate and running the Mef1 assay at optimal conditions for 2 h. St) Hydrolysate standard obtained after enzymatic reaction of FFA2 on Fe fucoidan.

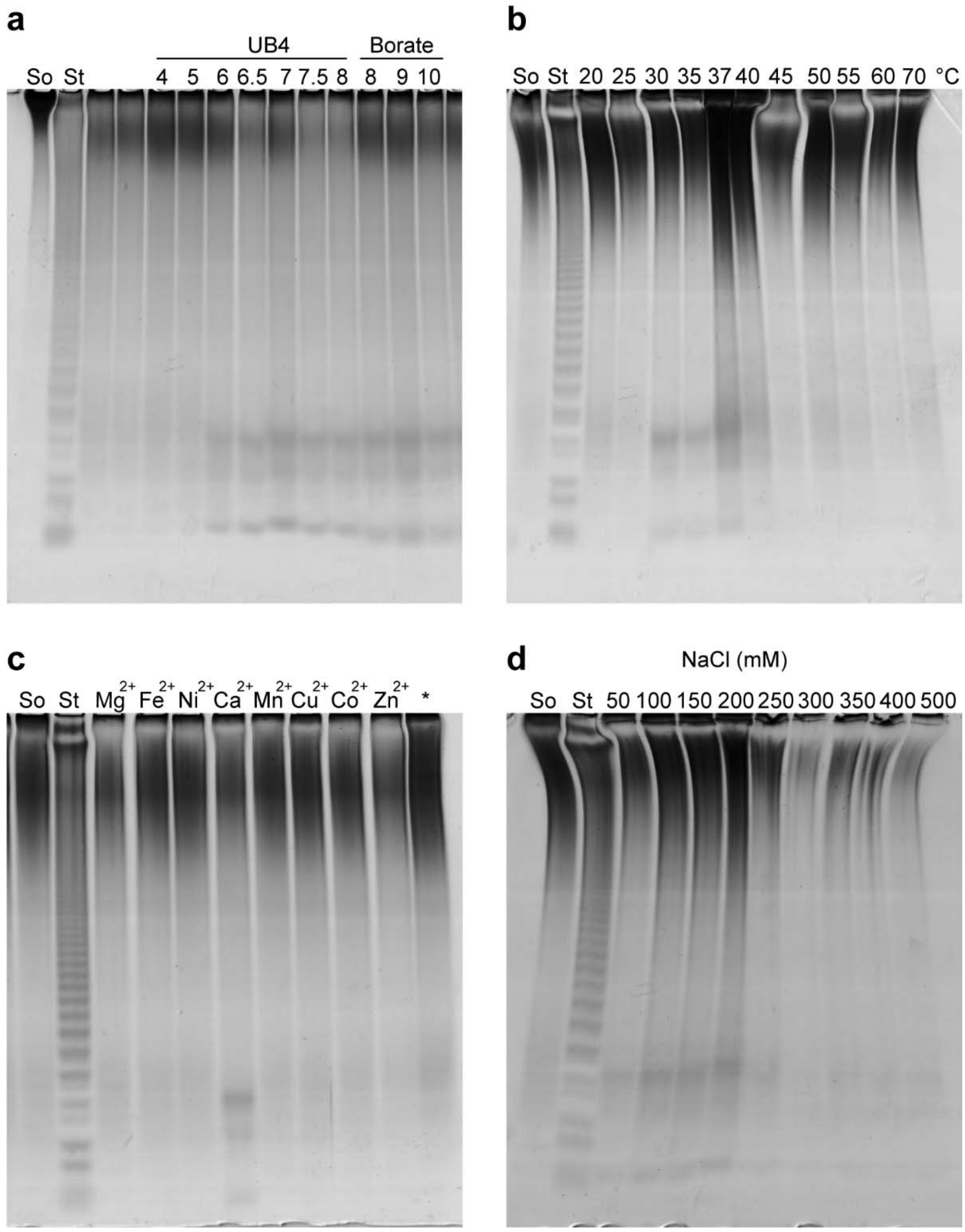

Figure S12 Characterization of Mef1 performance on the *S. oligocystum* substrate. The assay reaction included: Mef1 in 0.02 M Tris-HCl buffer, pH 9, 125 mM NaCl, 10 mM Ca^{2+}, and 0.9 %w/v fucoidan from *S. oligocystum* buffer. Data were run after reactions had run for 2 h at 37 °C.
